# Ferroptosis and macrophage polarization: mechanisms, interplay, and implications for medical applications

**DOI:** 10.1038/s41420-026-03147-2

**Published:** 2026-05-23

**Authors:** Yajuan Zhao, Jianbin Fu, Ping Zhao, Yuan Jiang, Yuhan Wang, Yaopeng Zhang, Zhaorui Li, Yongjun Zhao, Niannian Li, Bao Lang, Yanhua Luo

**Affiliations:** 1 Weifang People’s Hospital, Shandong Second Medical University, Weifang, China; 2 Tianjin Medical University, Tianjin, China; 3 Department of Anesthesiology, 80th Group Army Hospital of People’s Liberation Army, Weifang, China; 4 School of Anesthesiology, Shandong Second Medical University, Weifang, China

**Keywords:** Cell death and immune response, Cell death

## Abstract

Ferroptosis, a unique form of regulated cell death driven by iron-dependent lipid peroxidation, and macrophage polarization, which reflects the high plasticity of the immune system, represent two prominent frontiers in contemporary life sciences. Emerging evidence suggests a profound bidirectional interplay between these processes, mediated by iron metabolism reprogramming, lipid signaling molecules, and complex molecular axes such as RAGE-STAT3. This review systematically summarizes the biological mechanisms of ferroptosis and macrophage polarization, delving into their interaction within the tumor microenvironment, inflammatory responses, and cardiovascular and neurodegenerative diseases (e.g., Alzheimer’s and Parkinson’s). We highlight how M1 macrophages induce ferroptosis through pro-inflammatory cytokines and reactive oxygen species, while M2 macrophages inhibit it by modulating iron homeostasis and antioxidant capacity. Finally, we discuss therapeutic strategies targeting the ferroptosis-macrophage polarization axis, providing a theoretical foundation and novel perspectives for developing precise medical interventions.

## Facts

- Mechanistic Complexity and Context-Dependency: Although pathways like RAGE-STAT3 have been identified as bridges between ferroptosis and macrophage polarization, the precise molecular switches and uncharacterized signaling networks governing this interplay remain to be fully elucidated across different cell types and specific disease contexts.
- Exploration of Parallel Regulatory Pathways: Beyond the canonical GPX4-dependent axis, the role of non-canonical networks (such as the FSP1/NRF2 axis) in modulating macrophage susceptibility to ferroptosis is emerging as a critical research frontier for immune microenvironment remodeling.
- Metabolic Balance in the Tumor Microenvironment: While inducing ferroptosis in M2-like tumor-associated macrophages (TAMs) shows therapeutic promise, a key query remains: whether the metabolites released by ferroptotic TAMs inadvertently enhance the ferroptosis resistance of adjacent tumor cells.
- Challenges in Clinical Translation: Despite the potential of ferroptosis inducers and polarization modulators in animal models, achieving precise targeted delivery and ensuring long-term safety and efficacy in human patients remain the primary bottlenecks for clinical transition.
- From Single-Target to Combination Therapies: Given the multifactorial nature of complex pathologies, future research should leverage AI and multi-omics to develop personalized combination strategies that simultaneously calibrate both ferroptosis networks and macrophage functional states.

## Introduction

### Research background and significance

Ferroptosis and macrophage polarization have recently emerged as prominent research frontiers in the life sciences. Ferroptosis is a novel form of iron-dependent regulated cell death, characterized by iron-mediated lipid peroxidation driven by reactive oxygen species (ROS) generated via the Fenton reaction. It is distinctly different from traditional cell death modalities such as apoptosis, necrosis, and autophagy [1, 2]. Since its formal naming in 2012 [2], ferroptosis has rapidly become a focal point in scientific research, playing a crucial role in the pathogenesis and progression of various diseases including cancer, neurodegenerative disorders, and cardiovascular diseases. For instance, in cancer research, inducing ferroptosis in tumor cells is considered a highly promising anti-cancer strategy [3], as many tumor cells exhibit heightened sensitivity to ferroptosis. Regulating ferroptosis-related signaling pathways holds potential for effectively eliminating tumor cells [4–6]. In neurodegenerative diseases [7], such as Alzheimer’s disease and Parkinson’s disease, neuronal ferroptosis is recognized as a significant factor contributing to neuronal cell death and dysfunction. In-depth studies on the mechanisms of ferroptosis are expected to facilitate the development of novel neuroprotective therapeutic approaches.

Macrophage polarization is a key research topic in immunology. As essential components of the immune system, macrophages possess high plasticity and heterogeneity [8]. Under different microenvironmental stimuli, macrophages can polarize into distinct phenotypes, with the most representative being classically activated M1 macrophages and alternatively activated M2 macrophages [9, 10]. M1 macrophages play a pivotal role in inflammatory responses. They secrete large quantities of pro-inflammatory cytokines such as tumor necrosis factor-alpha (TNF-α), interleukin-1β (IL-1β), and interleukin-6 (IL-6), effectively activating immune responses and clearing pathogens. In contrast, M2 macrophages are primarily involved in immunoregulation, tissue repair, and tumor progression. They secrete cytokines like interleukin-10 (IL-10) and transforming growth factor-beta (TGF-β), which exert anti-inflammatory and tissue-repair functions. Within the tumor microenvironment, M2 macrophages often promote tumor growth, angiogenesis, and metastasis, significantly contributing to tumor immune escape [11].

Accumulating evidence indicates a close interplay between ferroptosis and macrophage polarization; these processes interact and jointly influence diverse physiological and pathological processes. Unraveling this connection provides a novel perspective for deepening our understanding of disease mechanisms [12, 13]. Within the tumor microenvironment (TME), the use of ferroptosis inducers can not only directly trigger ferroptosis in tumor cells but also modulate the polarization state of macrophages, thereby altering the immune landscape of the TME and enhancing the body’s immune surveillance and cytotoxic capabilities against tumors. Ferroptotic tumor cell death releases a series of damage-associated molecular patterns (DAMPs), which can be recognized by macrophages, subsequently influencing their polarization direction [14, 15]. A thorough understanding of the interaction mechanisms between ferroptosis and macrophage polarization would enable the precise regulation of this process, leading to the development of more effective disease treatment strategies. For cancer therapy, it is conceivable to design drugs or therapeutic regimens that, on one hand, induce ferroptosis in tumor cells [16, 17], and on the other hand, promote macrophage polarization towards the M1 phenotype, thereby enhancing anti-tumor immune responses for more effective tumor treatment [18]. In treating inflammatory diseases, modulating ferroptosis and macrophage polarization could help control the intensity and progression of inflammatory responses, promoting tissue repair and recovery [15, 18].

### Current research status on ferroptosis and macrophage polarization

In recent years, significant progress has been made in ferroptosis research, with increasingly in-depth exploration of its regulatory mechanisms. Glutathione peroxidase 4 (GPX4), acting downstream of the cystine/glutamate antiporter system xc − , has been identified as a key regulator of ferroptosis. It utilizes glutathione (GSH) to reduce peroxidized phospholipids to non-toxic phospholipid alcohols, thereby inhibiting ferroptosis. Inhibition of GPX4 activity or reduction in its expression levels leads to a sharp increase in intracellular lipid peroxidation, subsequently inducing ferroptosis [19, 20]. Iron metabolism-related proteins, such as transferrin receptor 1 (TFR1) [4], ferritin [21], and ferroportin (FPN) [22], also play important roles in ferroptosis. TFR1 is responsible for transporting iron ions into cells, ferritin stores iron ions, and FPN exports intracellular iron ions to the extracellular space. Dysregulated expression or dysfunction of these proteins can disrupt intracellular iron homeostasis, increasing cellular susceptibility to ferroptosis. Beyond iron metabolism and GPX4-related pathways, novel ferroptosis regulators and signaling pathways have been discovered. The ferroptosis suppressor protein 1 (FSP1)—coenzyme Q10 (CoQ10)—NAD(P)H pathway functions as an important inhibitory system independent of the GPX4-GSH axis. FSP1 reduces CoQ10 to ubiquinol, inhibiting lipid peroxidation and thereby suppressing ferroptosis [4, 21]. Certain metabolic pathways, such as the mevalonate pathway [23, 24] and fatty acid metabolism [25], are also closely linked to the regulation of ferroptosis.

Research on macrophage polarization has also yielded fruitful results, with continuously deepening understanding of its molecular mechanisms and functions. Signal transducer and activator of transcription (STAT) family proteins play a central role in macrophage polarization. During M1 macrophage polarization, interferon-gamma (IFN-γ) binding to its receptor on macrophages activates the JAK-STAT1 signaling pathway, inducing the expression of a series of M1-associated genes, such as inducible nitric oxide synthase (iNOS) and TNF-α. Conversely, during M2 macrophage polarization, interleukin-4 (IL-4) or interleukin-13 (IL-13) binding activates the JAK-STAT6 signaling pathway, promoting the expression of M2-associated genes like arginase 1 (Arg1) and mannose receptor (MR). Transcription factors such as nuclear factor-kappa B (NF-κB) and peroxisome proliferator-activated receptor-gamma (PPAR-γ) are also involved in regulating macrophage polarization. NF-κB is activated during M1 polarization, promoting pro-inflammatory gene expression, while PPAR-γ plays a significant role in M2 polarization, regulating genes related to anti-inflammatory responses and tissue repair.

Although significant progress has been made in the individual studies of ferroptosis and macrophage polarization, research on their interconnection remains in its early stages, with many gaps. The specific molecular mechanisms by which ferroptosis influences macrophage polarization, and how changes in macrophage polarization feedback to regulate the initiation and progression of ferroptosis, still lack comprehensive and systematic investigation. While some studies suggest that ferroptosis inducers can affect macrophage polarization direction, the involved signaling pathways and key molecules are not fully elucidated. Within the tumor microenvironment, how the dynamic balance between ferroptosis and macrophage polarization is maintained, and the impact of disrupting this balance on tumor development, progression, and therapeutic efficacy, require further exploration. In-depth research into the connection between ferroptosis and macrophage polarization holds significant theoretical importance and clinical application value for revealing disease pathogenesis and developing novel therapeutic targets and strategies. Therefore, this field offers vast research space and immense potential.

## Biological mechanisms of ferroptosis

### Definition and characteristics of ferroptosis

Ferroptosis is a regulated form of cell death characterized by iron-dependent accumulation of lipid peroxides, which drives a marked increase in intracellular reactive oxygen species (ROS), ultimately compromising plasma membrane integrity and leading to cell death. Ferroptosis exhibits several distinct differences from traditional cell death forms such as apoptosis, necrosis, and autophagy.

In terms of morphology, cells undergoing ferroptosis display unique changes. Mitochondria appear markedly shrunken with significantly increased membrane density, reduced or even disappeared cristae, and ruptured outer mitochondrial membranes. The overall cell size decreases, becoming more rounded, and intercellular connections gradually detach [2, 26]. In contrast, during apoptosis, cell volume shrinks, nuclear chromatin condenses and fragments, and the cell membrane blebs to form apoptotic bodies [27]; Necrosis is characterized by cell swelling and rapid plasma membrane rupture, releasing cellular contents. During autophagy, numerous autophagosomes form within the cell to encapsulate and degrade damaged organelles and proteins [27, 28].

Biochemically, the occurrence of ferroptosis is accompanied by specific changes, namely iron accumulation and lipid peroxidation [29]. Intracellular iron levels, particularly ferrous iron (Fe²⁺) in the labile iron pool (LIP), rise significantly. These iron ions can catalyze radical formation via the Fenton reaction, promoting lipid peroxidation [30–32]. During ferroptosis, the intracellular antioxidant system is severely compromised, with glutathione (GSH) levels drastically reduced and glutathione peroxidase 4 (GPX4) activity inhibited. GPX4 is a crucial antioxidant enzyme that uses GSH to reduce peroxidized phospholipids to non-toxic phospholipid alcohols, thus inhibiting ferroptosis. When GPX4 activity declines, lipid peroxidation products such as malondialdehyde (MDA) and 4-hydroxynonenal (4-HNE) accumulate extensively. These peroxidation products possess strong cytotoxicity, damaging plasma membrane structure and function, leading to cell death [26]. In apoptosis, the main biochemical feature is the activation of caspases, which cleave various intracellular proteins, altering cell structure and function. During necrosis, ATP levels rapidly drop, and intracellular ion balance is disrupted [33]; Autophagy involves the expression of autophagy-related proteins and the formation of autophagosomes [34].

Ferroptosis plays significant roles in various physiological and pathological processes. During tumorigenesis and progression, ferroptosis can act as a natural anti-cancer mechanism, limiting tumor cell growth and spread. Some tumor cells, due to their metabolic characteristics, are more sensitive to ferroptosis. Inducing ferroptosis in these cells presents a potential therapeutic approach. In neurodegenerative diseases like Alzheimer’s and Parkinson’s, neuronal ferroptosis is considered a major factor contributing to neuronal death and dysfunction. In cardiovascular diseases, ferroptosis occurs during ischemia-reperfusion injury, leading to cardiomyocyte and vascular endothelial cell death, exacerbating tissue damage. A deep understanding of the definition and characteristics of ferroptosis lays the foundation for further exploration of its role in disease mechanisms.

### Molecular regulatory mechanisms of ferroptosis

**Fig. 1:**
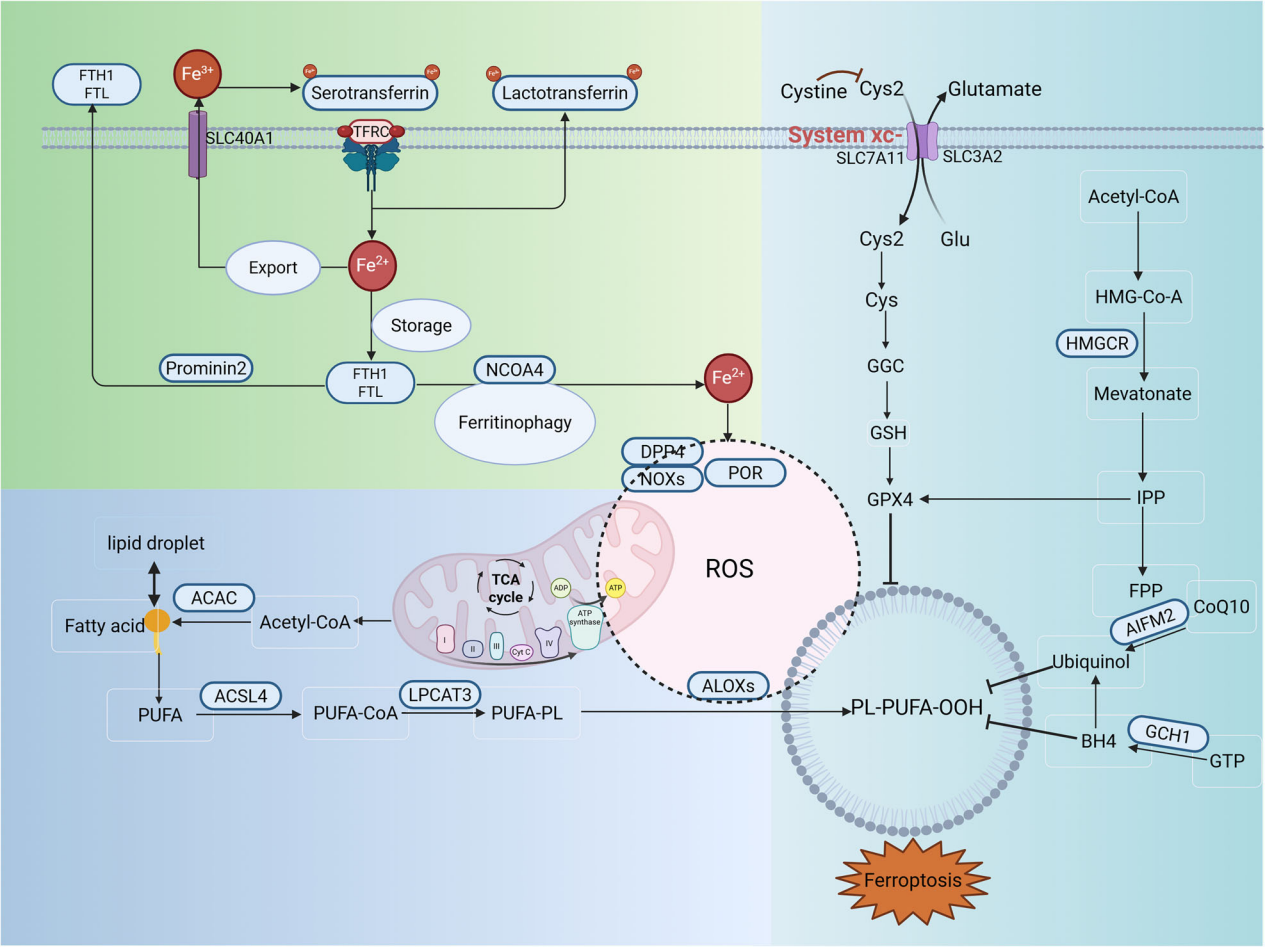
Mechanisms underlying ferroptosis. The process is mainly accompanied by the accumulation of iron and the formation of lipid peroxides, which eventually lead to cell death. This process of ferroptosis involves several pathways, such as System Xc-, iron metabolism and lipid metabolism.

The molecular regulatory mechanisms of ferroptosis constitute a complex and finely tuned process involving the interplay of multiple key molecules and signaling pathways. In-depth study of these regulatory mechanisms is crucial for elucidating the role of ferroptosis in physiological and pathological processes and for developing related therapeutic strategies (Fig. 1).

#### GPX4-related mechanisms

GPX4 occupies a central position in the intracellular antioxidant defense system. It is a selenoenzyme that utilizes GSH as a substrate to reduce peroxidized phospholipids (PL-PUFA-OOH) to non-toxic phospholipid alcohols (PL-PUFA-OH), effectively inhibiting lipid peroxidation and protecting cells from ferroptosis. In this process, the active site of GPX4 contains selenocysteine, which reacts with the peroxyl group of peroxidized phospholipids, reducing it to a hydroxyl group, while GSH is oxidized to glutathione disulfide (GSSG). GSSG can be reduced back to GSH by glutathione reductase (GR) using NADPH as a cofactor, maintaining intracellular GSH levels and ensuring sustained GPX4 activity [35].

Erastin and RSL3 are two classic ferroptosis inducers [33, 36], they inhibit GPX4 function through different mechanisms, leading to ferroptosis. Erastin primarily acts by inhibiting the cystine/glutamate antiporter System Xc− on the plasma membrane [37]. System Xc− is composed of SLC7A11 and SLC3A2 subunits. It transports extracellular cystine into the cell in a 1:1 exchange for intracellular glutamate. Intracellular cystine is reduced to cysteine, a critical precursor for GSH synthesis. When Erastin inhibits System Xc − , cystine uptake is blocked, intracellular cysteine levels decrease, leading to reduced GSH synthesis. The decline in GSH levels leaves GPX4 deprived of its substrate, unable to effectively reduce peroxidized phospholipids. Consequently, lipid peroxidation products accumulate, ultimately triggering ferroptosis [2].

RSL3 directly targets GPX4, binding to its active site and inhibiting its enzymatic activity. When RSL3 binds to GPX4, the enzyme cannot catalyze the reduction of peroxidized phospholipids normally. Intracellular lipid peroxidation levels rise rapidly, exceeding the cell’s antioxidant capacity, leading to ferroptosis. Although these two inducers have different primary mechanisms, both ultimately result in the inhibition of GPX4 function and the induction of ferroptosis. They serve as important tools for studying ferroptosis mechanisms and provide potential targets for developing ferroptosis-based therapies.

#### Mechanisms related to disrupted iron metabolism

Iron plays a crucial role in ferroptosis. Maintaining iron homeostasis is vital for normal cellular physiology. Intracellular iron primarily exists as Fe²⁺ in the labile iron pool (LIP). Fe²⁺ can directly catalyze radical formation via the Fenton reaction, a key step in ferroptosis initiation. In the Fenton reaction, Fe²⁺ reacts with hydrogen peroxide (H₂O₂), generating highly reactive hydroxyl radicals (·OH) and Fe³⁺. The hydroxyl radical is a potent oxidant that attacks polyunsaturated fatty acids (PUFAs) in cell membranes, initiating a lipid peroxidation chain reaction. In this chain reaction, the hydroxyl radical abstracts a hydrogen atom from a PUFA double bond, forming a lipid radical (L·). L· then reacts with oxygen to form a lipid peroxyl radical (LOO·). LOO· can abstract a hydrogen atom from another PUFA molecule, propagating the radical chain reaction and generating large amounts of lipid hydroperoxides (L-OOH). When lipid hydroperoxide accumulation surpasses the cell’s antioxidant capacity, plasma membrane integrity is compromised, and ferroptosis occurs [38].

Beyond promoting lipid peroxidation via the Fenton reaction, iron and its derivatives, such as heme or iron-sulfur [Fe-S] clusters, critically influence the activity of enzymes that generate ROS [39, 40]. Enzymes like NADPH oxidases (NOX), lipoxygenases (LOX), and mitochondrial electron transport chain complexes require iron or its derivatives as cofactors for their catalytic activity. NOX utilizes NADPH as an electron donor to reduce oxygen to superoxide anion (O₂⁻·), which can be further converted to other ROS like hydrogen peroxide and hydroxyl radical [41]. LOX catalyzes the oxidation of PUFAs, generating bioactive lipid hydroperoxides and lipid radicals. The mitochondrial electron transport chain also produces small amounts of ROS during electron transfer [42, 43]. When intracellular iron metabolism is dysregulated and iron levels are abnormally high, the activity of these enzymes increases, leading to excessive ROS production, further exacerbating lipid peroxidation and ferroptosis. The process of ferritinophagy is also closely related to ferroptosis. Ferritin is the primary intracellular iron storage protein, a complex composed of ferritin heavy chain (FTH1) and ferritin light chain (FTL) subunits, which sequesters iron ions to maintain iron homeostasis [44]. During ferritinophagy, ferritin is engulfed by autophagosomes, which then fuse with lysosomes. In the acidic lysosomal environment, ferritin is degraded, releasing stored iron ions. This increases the Fe²⁺ level in the intracellular LIP, promoting ferroptosis [2, 19, 45].

#### Other related mechanisms

Beyond GPX4 and iron metabolism-related mechanisms, the regulation of ferroptosis involves several other molecular pathways that interact to influence its initiation and progression.

Amino acid metabolism pathways play important roles in regulating ferroptosis. Cysteine, as the rate-limiting substrate for GSH synthesis, critically impacts cellular sensitivity to ferroptosis. As mentioned, cystine enters cells via System Xc− and is reduced to cysteine, which participates in GSH synthesis, thereby affecting GPX4 activity. Glutamine metabolism is also closely linked to ferroptosis. Glutamine is converted to glutamate by glutaminase (GLS). Glutamate not only participates in System Xc− transport but also serves as a precursor for synthesizing other amino acids and biomolecules. In some tumor cells, glutamine metabolism is abnormally active, providing energy and biosynthetic precursors for proliferation and survival. Inhibition of glutamine metabolism can compromise cellular energy and precursor supply, affecting antioxidant capacity and increasing sensitivity to ferroptosis [46]. Certain enzymes and transporters related to amino acid metabolism, such as arginase 1 (Arg1) and cationic amino acid transporter 2 (CAT2), have also been implicated in ferroptosis regulation. Arg1 converts arginine to ornithine and urea, regulating intracellular arginine levels and thereby influencing cell metabolism and function. CAT2 is responsible for transporting cationic amino acids like arginine into cells. Alterations in its expression or function affect the uptake and utilization of these amino acids, influencing ferroptosis [47, 48].

Lipid metabolism pathways are also integral components of ferroptosis regulation. Lipid peroxidation is the hallmark event of ferroptosis, and processes involved in lipid synthesis, metabolism, and modification all influence its occurrence. Acyl-CoA synthetase long-chain family member 4 (ACSL4) and lysophosphatidylcholine acyltransferase 3 (LPCAT3) play key roles in lipid peroxidation [49, 50]. ACSL4 catalyzes the conjugation of long-chain polyunsaturated fatty acids (PUFAs) to coenzyme A (CoA), forming PUFA-CoA esters. LPCAT3 facilitates the esterification and incorporation of PUFA-CoA esters into membrane phospholipids, enriching membranes with PUFAs. These PUFA-rich membrane phospholipids, due to their structural characteristics, are susceptible to ROS attack, undergoing lipid peroxidation and promoting ferroptosis [51]. Other enzymes and signaling pathways related to lipid metabolism, such as phospholipase A2 (PLA2) and protein kinase C (PKC), also participate in ferroptosis regulation [43]. PLA2 hydrolyzes membrane phospholipids, releasing PUFAs like arachidonic acid, providing substrates for lipid peroxidation [25]. PKC can regulate the activity of proteins involved in lipid metabolism and ferroptosis, such as ACSL4 and GPX4, through phosphorylation, thereby influencing ferroptosis [52].

## Biological mechanisms of macrophage polarization

### Overview of macrophages

Macrophages are indispensable members of the immune system, playing crucial roles in immune defense, immunoregulation, and the maintenance of tissue homeostasis. Macrophages originate from hematopoietic stem cells in the bone marrow. These stem cells first differentiate into monocytes, which enter the bloodstream and further mature into macrophages within different tissue microenvironments. Macrophages are widely distributed throughout various tissues and organs, assuming different names and functional characteristics. In the liver, they are called Kupffer cells, efficiently clearing pathogens, senescent cells, and foreign particles from the blood to maintain normal liver function. In the lungs, alveolar macrophages phagocytose inhaled dust, bacteria, and viruses, protecting the respiratory tract from infection. In the nervous system, microglia, as specialized macrophages, not only participate in immune defense but also play vital supportive roles in neuronal development, maintenance, and repair. Macrophages are key players in immunoregulation. They can act as antigen-presenting cells, processing and presenting ingested pathogen antigens to T lymphocytes, thereby activating adaptive immune responses. Macrophages also secrete various cytokines and chemokines, such as tumor necrosis factor-alpha (TNF-α), interleukin-1 (IL-1), interleukin-6 (IL-6), and monocyte chemoattractant protein-1 (MCP-1). These factors regulate the activity and function of other immune cells, promoting or suppressing inflammatory responses to maintain immune balance. Macrophages are also essential for maintaining tissue homeostasis. They clear senescent cells, apoptotic cells, and cellular debris from the body, keeping tissues clean and healthy [53, 54]. Additionally, macrophages secrete growth factors and extracellular matrix components, promoting tissue repair and regeneration. During wound healing, macrophages secrete various growth factors like platelet-derived growth factor (PDGF) and transforming growth factor-beta (TGF-β), stimulating fibroblast proliferation and collagen synthesis to facilitate wound closure [53, 55, 56]. As a vital component of the immune system, the diversity in origin, distribution, and function of macrophages underscores their irreplaceable role in maintaining health and combating disease.

### Types and characteristics of macrophage polarization

**Fig. 2:**
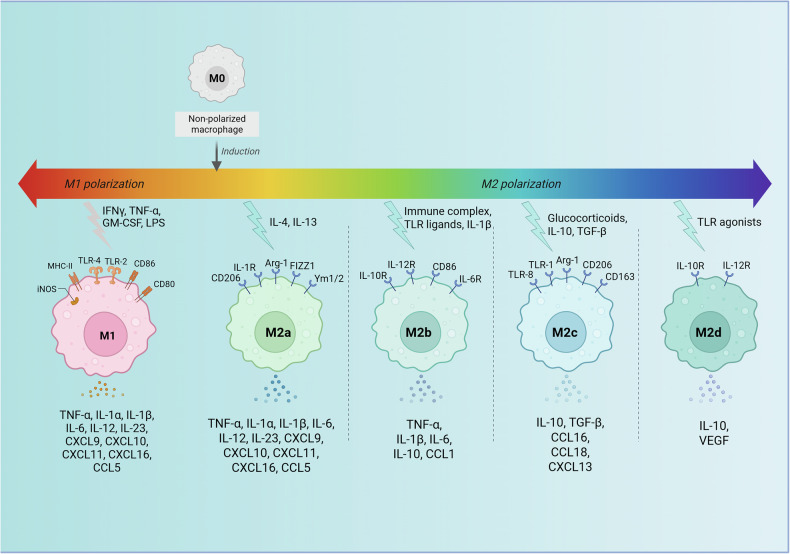
Spectrum of macrophage polarization from M0 to M1 and M2 subtypes. There are two main forms of polarization of macrophages, M1-like and M2-like, which can be polarized into two types, respectively, under different cell stimulation conditions. M2-like macrophages can also be divided into four subtypes, M2a, M2b, M2c and M2d. Different macrophages have different markers on their surface, which can be used to identify the difference. The two types of macrophages secrete different cytokines during the disease process, resulting in two opposite effects.

Macrophage polarization refers to the process by which macrophages acquire different functional and phenotypic states in response to various microenvironmental stimuli. This process is crucial for immune responses, tissue repair, inflammation, and the pathogenesis of numerous diseases. Under physiological and pathological conditions, macrophages primarily polarize into two main subtypes: M1 and M2. These subtypes exhibit significant differences in inducing conditions, surface markers, secreted cytokines, and functions [57] (Fig. 2).

M1 macrophages, also known as classically activated macrophages, are primarily activated in the context of Th1-type immune responses and acute inflammation. When the body is infected by

pathogens like bacteria or viruses, pathogen-associated molecular patterns (PAMPs), such as lipopolysaccharide (LPS), bind to Toll-like receptors (TLRs) on macrophages, activating downstream signaling pathways and inducing M1 polarization [58]. Interferon-gamma (IFN-γ) secreted by type 1 T helper (Th1) cells also strongly promotes M1 polarization. M1 macrophages highly express surface markers like major histocompatibility complex class II (MHC II) and co-stimulatory molecules CD80 and CD86. These markers aid in antigen presentation to T cells, activating T cell immune responses [59]. M1 macrophages possess potent bactericidal and anti-tumor capabilities. They secrete large amounts of pro-inflammatory cytokines such as TNF-α, IL-1β, IL-6, and IL-12 [54, 60]. These cytokines activate other immune cells, enhancing the body’s immune defense and promoting inflammatory responses to effectively eliminate pathogens and tumor cells. M1 macrophages also produce substantial amounts of nitric oxide (NO), which has strong cytotoxicity and can directly kill pathogens and tumor cells [61].

M2 macrophages, or alternatively activated macrophages, primarily function in Th2-type immune responses, tissue repair, and immunoregulation. Interleukin-4 (IL-4) and interleukin-13 (IL-13) are key cytokines inducing M2 polarization, secreted mainly by Th2 cells, eosinophils, and mast cells. During parasitic infections, allergic reactions, and wound healing, levels of IL-4 and IL-13 increase, promoting M2 polarization [56, 62]. M2 macrophages express specific surface markers such as mannose receptor (CD206), arginase 1 (Arg1), and CD163 [63]. These markers are closely related to M2 functions. CD206 recognizes and binds mannose residues on pathogen surfaces, promoting phagocytosis and clearance. Arg1 metabolizes arginine to ornithine and urea, participating in tissue repair and cell proliferation. CD163 is a scavenger receptor that clears hemoglobin-haptoglobin complexes from circulation, exerting anti-inflammatory and immunoregulatory effects [64]. Cytokines secreted by M2 macrophages are primarily anti-inflammatory, such as IL-10 and TGF-β. IL-10 inhibits the activity of other immune cells, reducing pro-inflammatory cytokine production, thus exerting anti-inflammatory effects. TGF-β promotes fibroblast proliferation and collagen synthesis, aiding tissue repair and fibrosis [65]. M2 macrophages also participate in immunoregulation by inhibiting T cell activation and proliferation, maintaining immune homeostasis, and preventing excessive immune responses that could harm the host [66].

M1 and M2 macrophages represent two extreme phenotypes of the polarization spectrum. In actual physiological and pathological contexts, macrophage polarization states exist on a continuum with numerous intermediate states. Their function and phenotype dynamically adjust according to microenvironmental changes. Research on the types and characteristics of macrophage polarization provides a crucial theoretical foundation for understanding immune system regulation and disease pathogenesis.

### Molecular regulatory mechanisms of macrophage polarization

**Fig. 3:**
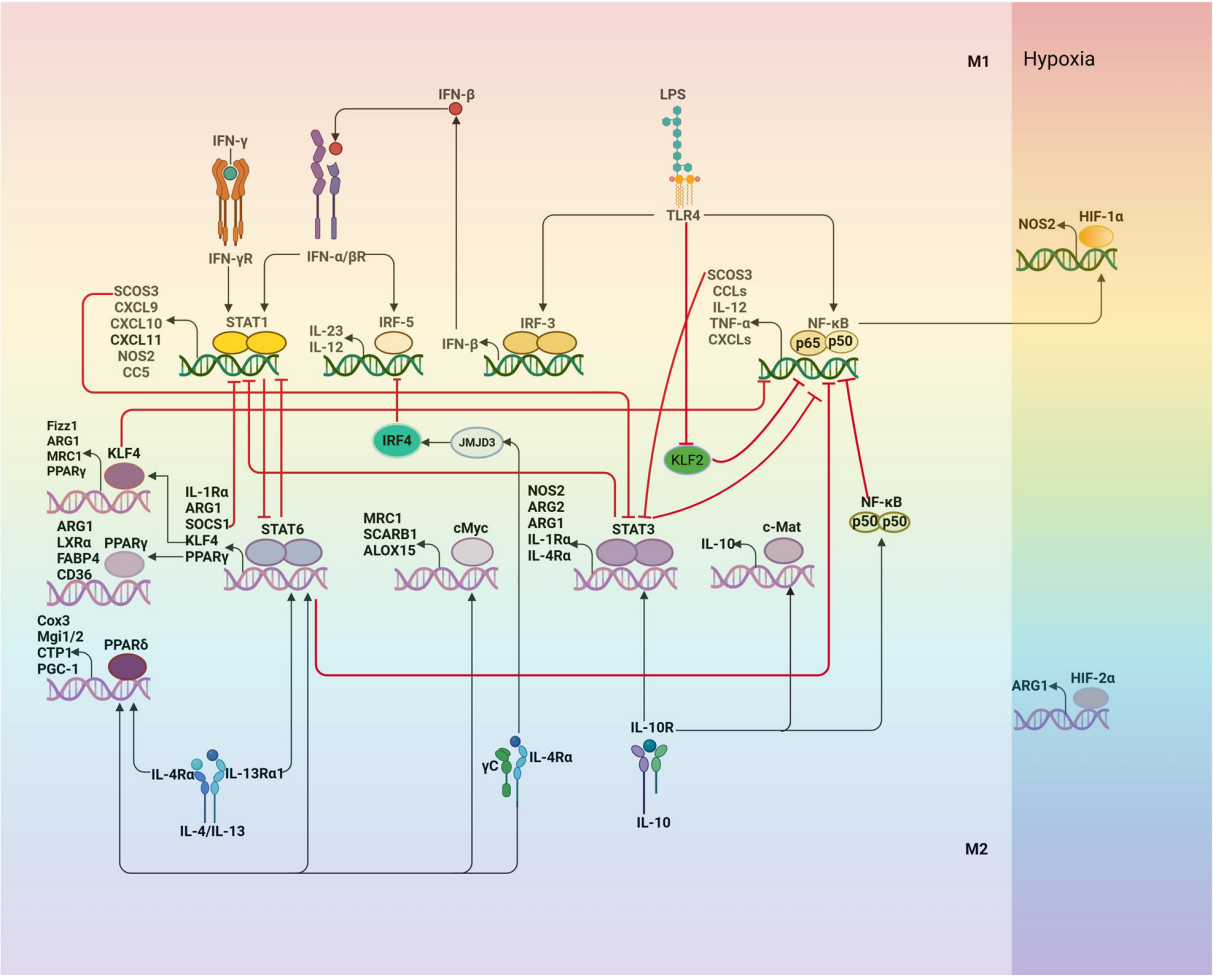
Mechanisms of macrophage polarization. This section outlines the major pathways governing macrophage polarization and highlights the crosstalk between M1 and M2-polarizing signals. The balance between STAT1 and STAT3/STAT6 activation serves as a central regulatory node, finely tuning macrophage polarization and functional output. Predominant activation of NF-κB and STAT1 drives M1 polarization, leading to macrophages with cytotoxic and pro-inflammatory functions. Conversely, dominant activation of STAT3 and STAT6 promotes M2 polarization, which is associated with immunosuppressive functions and tumor progression. Specific regulators further define M2 phenotypes: PPARγ and PPARδ govern distinct aspects of M2 activation and oxidative metabolism; KLF4 and KLF2 promote M2 functions by cooperating with STAT6 and suppressing NF-κB-/HIF-1α-dependent transcription, respectively; and IL-4-induced c-Myc activity regulates a subset of M2-associated genes. Furthermore, IL-4 reinforces M2 commitment through the Jmjd3-IRF4 axis, which inhibits IRF5-mediated M1 polarization. IL-10 also promotes M2 polarization by inducing the p50 NF-κB homodimer, c-Maf, and STAT3 activities.

The molecular regulatory mechanisms of macrophage polarization constitute a complex and precise process involving the coordinated action of multiple signaling pathways and transcription factors (Fig. 3). Dysregulation of these mechanisms is closely associated with the pathogenesis of various diseases. In-depth study of these molecular mechanisms is crucial for elucidating disease pathogenesis and developing novel therapeutic strategies.

#### Regulation by signaling pathways

Multiple signaling pathways play key regulatory roles in macrophage polarization, with the nuclear factor-kappa B (NF-κB) and Janus kinase-signal transducer and activator of transcription (JAK-STAT) pathways being the most prominent.

The NF-κB signaling pathway plays a central role in M1 macrophage polarization [67]. When macrophages are stimulated by agents like LPS or TNF-α, surface receptors such as TLRs or TNFRs are activated, recruiting a series of adaptor proteins and kinases to form a signaling complex [68]. Within this complex, IκB kinase (IKK) is activated, phosphorylating IκB proteins, leading to their degradation and the release of NF-κB dimers [69]. The NF-κB dimers then translocate to the nucleus, bind to κB sites in the promoter regions of target genes, and initiate the transcription of M1-associated genes such as iNOS, IL-1β, IL-6, and TNF-α. The products of these genes promote inflammation and enhance the bactericidal and anti-tumor capacities of macrophages. Studies show that inhibiting NF-κB activity in LPS-stimulated macrophages significantly reduces the expression of M1 markers like iNOS and IL-1β, suppressing M1 polarization [70, 71].

The JAK-STAT signaling pathway also plays a crucial role, differentially regulating M1 and M2 polarization [69, 72]. In M1 polarization, IFN-γ binding to its receptor induces receptor dimerization and activation of associated JAK1 and JAK2 kinases. Activated JAKs phosphorylate tyrosine residues on the receptor, creating docking sites for STAT1. STAT1 is recruited, phosphorylated by JAKs, forms homodimers, translocates to the nucleus, and binds to gamma-activated sequence (GAS) elements in target gene promoters, promoting the expression of M1-associated genes like iNOS and IL-12 [73]. In M2 polarization, IL-4 or IL-13 binding to the IL-4 receptor alpha chain (IL-4Rα) activates JAK1 and JAK3. Activated JAKs phosphorylate IL-4Rα, recruiting and phosphorylating STAT6 [74]. Phosphorylated STAT6 forms homodimers, enters the nucleus, and binds specific sequences in promoter regions, inducing the expression of M2-associated genes like Arg1 and CD206. Gene knockout studies have shown that STAT1 deletion impairs macrophage response to IFN-γ and M1 polarization, while STAT6 deletion inhibits IL-4-induced M2 polarization [75].

Beyond NF-κB and JAK-STAT, other pathways like the mitogen-activated protein kinase (MAPK) and phosphatidylinositol 3-kinase-protein kinase B (PI3K-Akt) pathways also participate in macrophage polarization regulation. These pathways interact, forming a complex signaling network that collectively regulates the polarization process [76, 77].

#### Regulation by transcription factors

Transcription factors play pivotal roles in regulating the expression of genes associated with macrophage polarization. They bind to specific sequences in gene promoter regions, modulating transcriptional activity and thereby determining macrophage polarization direction and functional phenotype [78]. Interferon regulatory factor 5 (IRF5) and signal transducer and activator of transcription 6 (STAT6) are two representative transcription factors.

IRF5 plays a significant regulatory role in M1 macrophage polarization [79, 80]. Upon stimulation with LPS or IFN-γ, IRF5 is activated and phosphorylated. Phosphorylated IRF5 translocates from the cytoplasm to the nucleus, binds to specific sequences in the promoters of M1-associated genes, and promotes their transcription [81]. IRF5 can synergize with NF-κB to enhance the expression of M1 genes like IL-1β, IL-6, and TNF-α [82]. Studies find that macrophages from IRF5 knockout mice show significantly reduced expression of M1-associated genes and diminished pathogen-killing capacity [83]. IRF5 can also indirectly influence polarization by regulating the activity of other transcription factors [81, 84].

STAT6 is a core regulator of M2 macrophage polarization. As mentioned, IL-4 or IL-13 stimulation activates the JAK-STAT6 pathway, leading to STAT6 phosphorylation, dimerization, and nuclear translocation. In the nucleus, STAT6 binds to specific sequences in M2-associated gene promoters, promoting their transcription [78, 85]. STAT6 regulates the expression of M2 markers like Arg1, CD206, and Ym1, whose products are involved in M2 functions like immunoregulation and tissue repair [86, 87]. Experimental inhibition of STAT6 activity significantly suppresses M2 polarization and reduces the secretion of anti-inflammatory cytokines like IL-10 and TGF-β. STAT6 can also interact with other transcription factors to co-regulate M2 gene expression [63].

Besides IRF5 and STAT6, numerous other transcription factors participate, including NF-κB, peroxisome proliferator-activated receptor-gamma (PPAR-γ), and STAT1 [88–91]. These factors cooperate or compete, forming a complex transcriptional regulatory network that precisely controls the expression of polarization-related genes, determining macrophage polarization state and function.

## Interactions between ferroptosis and macrophage polarization

Ferroptosis and macrophage polarization, as significant research areas in cell biology and immunology, exhibit complex and intimate interactions. These interactions play critical roles in both physiological and pathological processes. Delving into their relationship is essential for elucidating the mechanisms of various diseases and developing novel therapeutic strategies.

### Impact of ferroptosis on macrophage polarization

#### Ferroptosis induces changes in macrophage iron metabolism

During ferroptosis, significant alterations in intracellular iron metabolism profoundly influence macrophage polarization. Under normal physiological conditions, macrophages employ various mechanisms to maintain iron homeostasis for proper function. Macrophages uptake transferrin-bound iron via transferrin receptor 1 (TFR1). Intracellular ferritin stores excess iron, and ferroportin (FPN) exports iron out of the cell. When ferroptosis occurs, this iron metabolic balance is disrupted, leading to iron overload [7].

Macrophages are central to systemic iron metabolism, recycling iron from phagocytosed senescent red blood cells, constituting a major source of bioavailable iron. Iron overload significantly impacts macrophage polarization. Substantial evidence indicates that high iron concentrations stimulate macrophages to secrete pro-inflammatory cytokines, increasing their propensity to polarize towards the M1 phenotype [92–94]. In inflammatory contexts, iron-laden macrophages highly express inducible nitric oxide synthase (iNOS), producing large amounts of nitric oxide (NO), and secrete pro-inflammatory cytokines like IL-1β, IL-6, and TNF-α [95]. These cytokines activate other immune cells, amplifying inflammatory responses for immune defense functions like antimicrobial and anti-tumor activity. Iron overload can also activate the nuclear factor-kappa B (NF-κB) signaling pathway, promoting the expression of M1-associated genes. Under iron overload, intracellular Fe²⁺ may interact with key proteins in the NF-κB pathway, promoting its activation and nuclear translocation. NF-κB then binds to κB sites in M1 gene promoters, initiating transcription and further driving M1 polarization [96].

#### Ferroptosis influences macrophage polarization via other metabolic reprogramming

Beyond changes in iron metabolism, ferroptosis triggers the reprogramming of several other metabolic pathways within macrophages, which also importantly regulate polarization.

##### Lipid metabolism pathways

These pathways play key roles in both ferroptosis and macrophage polarization. The hallmark of ferroptosis is lipid peroxide accumulation, and macrophage polarization states are closely linked to lipid metabolism. During ferroptosis, altered activity of key enzymes like acyl-CoA synthetase long-chain family member 4 (ACSL4) and lysophosphatidylcholine acyltransferase 3 (LPCAT3) increases the polyunsaturated fatty acid (PUFA) content in cell membranes, making them more sensitive to oxidative stress and prone to lipid peroxidation. ACSL4 catalyzes PUFA conjugation to CoA, forming PUFA-CoA esters. LPCAT3 facilitates the esterification and incorporation of these esters into membrane phospholipids. These PUFA-rich phospholipids are easily oxidized by ferroptosis inducers, forming lipid peroxidation products like malondialdehyde (MDA) and 4-hydroxynonenal (4-HNE) [97–99]. Accumulation of these products not only damages membrane integrity and function but can also act as signaling molecules influencing macrophage polarization [100–102]. Studies show that lipid peroxidation products can activate signaling pathways in macrophages, such as mitogen-activated protein kinase (MAPK) and NF-κB pathways, promoting M1 polarization. Activation of these pathways induces the expression of M1-associated genes like iNOS, IL-1β, and TNF-α, enhancing pro-inflammatory functions [103].

Amino Acid Metabolism Alterations are another important route through which ferroptosis affects polarization. Cysteine, as the rate-limiting substrate for GSH synthesis, critically impacts both ferroptosis sensitivity and macrophage polarization. During ferroptosis, inhibition of System Xc impedes cystine uptake, lowering intracellular cysteine and subsequently GSH levels. The reduction in GSH not only weakens antioxidant defenses, promoting ferroptosis, but also affects macrophage polarization. Research indicates that decreased GSH levels can disrupt intracellular redox balance, activating oxidative stress-related pathways that promote M1 polarization [97, 98]. Glutamine metabolism is also closely tied to both processes. Glutamine is converted to glutamate by glutaminase (GLS). Glutamate participates in System Xc− transport and serves as a precursor for other biomolecules. Alterations in glutamine metabolism during ferroptosis affect cellular energy and precursor supply, thereby influencing polarization. Studies find that inhibiting glutamine metabolism reduces ATP levels in macrophages, leading to energy insufficiency. This can suppress M2 polarization (which requires more energy for immunoregulatory and reparative functions) and promote a shift towards the more energy-efficient, defense-oriented M1 phenotype [99, 104–106].

Increasing research evidence demonstrates that non-GPX4-dependent ferroptosis pathways also play crucial roles within macrophages, profoundly regulating their functional diversity and dynamic responses to environmental stimuli. For instance, in apical periodontitis (AP) models, the transcription factor NRF2 has been shown to regulate macrophage ferroptosis specifically through the NRF2-FSP1-ROS axis, thereby facilitating bone loss in AP [107]. Furthermore, macrophage ferroptosis plays a pivotal role in the development of atherosclerosis (AS), a pathological process predominantly associated with the NRF2 regulatory network. Recent studies have provided novel mechanistic insights into AS-targeted therapies, demonstrating that Micheliolide (MCL) can attenuate atherosclerosis by competitively binding to the Arg483 site of KEAP1. This precise interaction disrupts the KEAP1/NRF2 complex, thereby suppressing macrophage ferroptosis and consequently limiting plaque progression [108]. Additionally, alongside the FSP1 pathway, other complex regulatory networks coordinate macrophage susceptibility to ferroptosis. For example, research has indicated that cigarette tar promotes atherosclerosis progression by inducing macrophage ferroptosis via the NF-κB-activated hepcidin/FPN/SLC7A11 pathway [109]. Collectively, these findings highlight that beyond the canonical GPX4 axis, a sophisticated network of parallel pathways actively governs macrophage ferroptosis across various disease contexts.

### Impact of macrophage polarization on ferroptosis

#### M1 macrophages and ferroptosis

M1 macrophages, as classically activated cells, play key roles in inflammation and immune defense. The substances they produce significantly induce or inhibit ferroptosis.

M1 macrophages secrete abundant cytokines that play important roles in regulating ferroptosis. Tumor necrosis factor-alpha (TNF-α), a major pro-inflammatory cytokine from M1 cells, can induce ferroptosis through multiple pathways [110]. TNF-α can activate receptor-interacting protein kinase 1 (RIPK1) and RIPK3, forming a necrosome that activates mixed lineage kinase domain-like protein (MLKL), leading to plasma membrane damage and cell death [111]. In this process, RIPK3 phosphorylates MLKL, causing its oligomerization and translocation to the plasma membrane to form pores, resulting in cell lysis. TNF-α can also activate the NF-κB pathway, promoting the expression of ferroptosis-related genes like transferrin receptor 1 (TFR1) [112, 113]. Increased TFR1 expression enhances cellular iron uptake, promoting ferroptosis. Interleukin-1β (IL-1β), another pro-inflammatory cytokine from M1 cells, can activate the NOD-like receptor protein 3 (NLRP3) inflammasome, promoting cytokine release and inflammation [114]. NLRP3 inflammasome activation leads to caspase-1 activation, which cleaves pro-IL-1β into its mature, secreted form [115]. IL-1β can act on neighboring cells in a paracrine manner, regulating their iron metabolism and oxidative stress levels, thereby influencing ferroptosis [116].

Reactive oxygen species (ROS) produced by M1 macrophages also play a key role in ferroptosis [117]. Upon activation, M1 macrophages exhibit enhanced respiratory burst activity, generating large amounts of ROS like superoxide anion (O₂⁻·), hydrogen peroxide (H₂O₂), and hydroxyl radical (·OH). These ROS can directly attack membrane PUFAs, initiating lipid peroxidation chain reactions that damage membranes and induce ferroptosis. ROS can also regulate the expression and activity of iron metabolism proteins, affecting iron homeostasis and promoting ferroptosis. Studies indicate that ROS can oxidize iron regulatory proteins (IRPs), enhancing their binding to iron-responsive elements (IREs). This increases TFR1 expression (promoting iron uptake) and suppresses ferritin expression (reducing iron storage), leading to elevated intracellular iron levels and promoting ferroptosis [118].

M1 macrophages can also regulate ferroptosis via nitric oxide (NO) secretion. NO is generated by inducible nitric oxide synthase (iNOS) in M1 macrophages from L-arginine. NO has diverse biological functions. In ferroptosis regulation, NO can react with superoxide anion to form peroxynitrite (ONOO⁻), a strong oxidant that directly oxidizes membrane lipids and proteins, causing damage and ferroptosis [119]. NO can also inhibit mitochondrial respiratory chain complexes I and II, reducing ATP production, causing energy metabolism dysfunction, and promoting ferroptosis [120]. Under certain conditions, NO may also inhibit lipid peroxidation, exerting a protective effect against ferroptosis, possibly by reacting with lipid radicals and terminating chain reactions [103].

#### M2 macrophages and ferroptosis

M2 macrophages, as alternatively activated cells, play important roles in immunoregulation, tissue repair, and tumor progression. Their unique roles in iron metabolism regulation and antioxidant capacity significantly influence ferroptosis.

M2 macrophages have distinct functions in iron metabolism regulation, importantly affecting ferroptosis. M2 macrophages highly express proteins related to iron uptake and storage, such as transferrin receptor 1 (TFR1), ferritin, and ferroportin (FPN). Changes in the expression of these proteins can modulate intracellular iron levels, influencing ferroptosis sensitivity [121]. While M2 cells express high levels of TFR1, facilitating iron uptake, they also highly express ferritin, which sequesters the acquired iron, reducing Fe²⁺ levels in the labile iron pool. Ferritin, a hollow spherical protein composed of 24 subunits, stores iron within its cavity, lowering iron reactivity and its participation in Fenton reactions, thereby inhibiting lipid peroxidation and ferroptosis. M2 macrophages also highly express FPN, which exports intracellular iron, further lowering intracellular iron concentration and reducing ferroptosis [122]. Studies in inflammatory models show that M2 macrophages, by regulating iron metabolism proteins, effectively lower intracellular iron levels, inhibit ferroptosis, and promote tissue repair and inflammation resolution [123].

The strong antioxidant capacity of M2 macrophages is another key factor influencing ferroptosis. Anti-inflammatory cytokines secreted by M2 cells, such as interleukin-10 (IL-10) and transforming growth factor-beta (TGF-β), play crucial roles in combating oxidative stress. IL-10 can inhibit the activity of other immune cells, reducing the production of pro-inflammatory cytokines and ROS, thereby lowering oxidative stress and inhibiting ferroptosis [123]. IL-10 can suppress M1 macrophage activation, reducing their secretion of TNF-α and IL-1β, which in turn decreases ROS production induced by these cytokines [124–126]. IL-10 can also directly act on target cells, upregulating the expression of antioxidant enzymes like superoxide dismutase (SOD), catalase (CAT), and glutathione peroxidase (GPX), enhancing antioxidant defenses and inhibiting ferroptosis. TGF-β has similar effects. It promotes the proliferation of cells like fibroblasts and collagen synthesis while inhibiting inflammation and oxidative stress, reducing ferroptosis [127]. TGF-β can activate the Smad signaling pathway, regulating the expression of antioxidant enzymes and iron metabolism proteins, thus inhibiting ferroptosis [128]. In tissue injury models, IL-10 and TGF-β secreted by M2 macrophages effectively suppress oxidative stress and ferroptosis, promoting tissue repair and regeneration [129].

### Molecular mechanisms of the interaction between ferroptosis and macrophage polarization

**Fig. 4:**
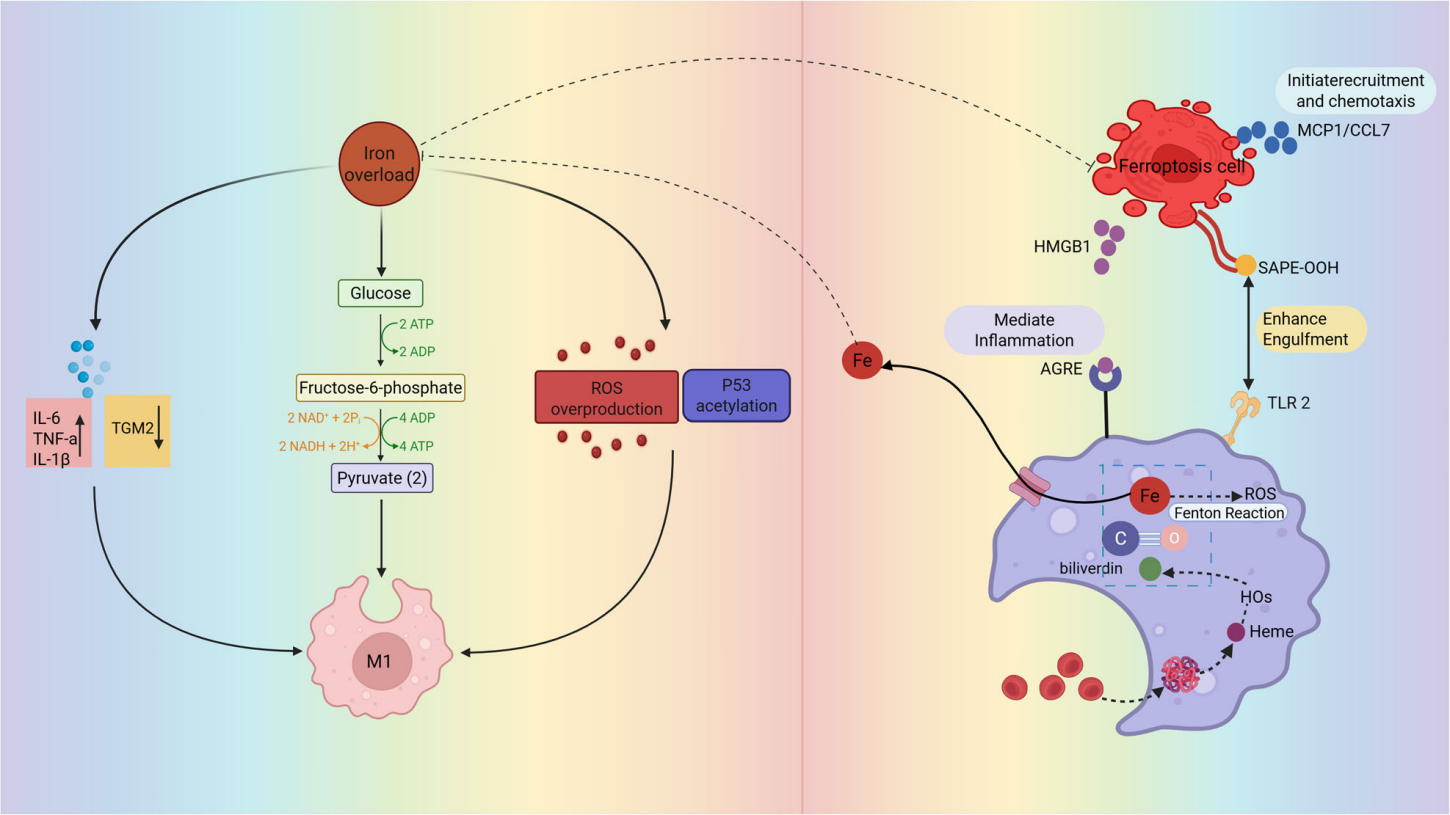
Interaction between macrophages and ferroptosis. Macrophages engulf red blood cells (RBCs) and digest them into hemoglobin, which is further broken down into heme. Heme is subsequently degraded into biliverdin, carbon monoxide, and iron. The iron derived from heme can either promote ROS production and lipid peroxidation or be released into the extracellular environment via ferroportin (FPN), thereby elevating iron levels both intracellularly in macrophages and in the surrounding tissue. In response to ferroptotic cells, macrophage Toll-like receptor 2 (TLR2) recognizes and binds to phosphatidylserine oxidized by lipid peroxides (SAPE-OOH) on the surface of ferroptotic cells, enhancing their phagocytic clearance. Additionally, high mobility group box 1 (HMGB1) released by dying cells interacts with the receptor for advanced glycation end products (RAGE) on macrophages, mediating inflammatory responses. Chemokines such as CCL2 and CCL7 further recruit macrophages and promote chemotaxis, amplifying immune reactions. Iron accumulation within macrophages drives M1 polarization through three primary mechanisms: upregulation of M1 markers (e.g., IL-6, TNF-α, IL-1β) and downregulation of M2 markers (e.g., TGM2); enhancement of glycolysis favoring an M1 phenotype; and induction of ROS elevation and p53 acetylation, which collectively promote an M1-like macrophage state.

The interaction between ferroptosis and macrophage polarization involves multiple complex molecular mechanisms (Fig. 4). The receptor for advanced glycation end products (RAGE/AGER) plays an important role in this interplay. During ferroptosis, cells release damage-associated molecular patterns (DAMPs), including advanced glycation end products (AGEs). AGEs can bind to RAGE on macrophage surfaces, activating downstream signaling. Research indicates that RAGE activation promotes macrophage uptake and processing of material released by ferroptotic cells, subsequently influencing macrophage polarization [130]. In the tumor microenvironment, ferroptotic tumor cells release AGEs. These AGEs bind to RAGE on tumor-associated macrophages (TAMs), activating intracellular signaling and promoting TAM polarization towards the M2 phenotype. In this process, RAGE activation leads to phosphorylation and activation of STAT3 [131].

Macrophages engulf red blood cells (RBCs) and digest them into hemoglobin, which is further broken down into heme. Heme is subsequently degraded into biliverdin, carbon monoxide, and iron. The iron derived from heme can either promote ROS production and lipid peroxidation or be released into the extracellular environment via ferroportin (FPN), thereby elevating iron levels both intracellularly in macrophages and in the surrounding tissue. In response to ferroptotic cells, macrophage Toll-like receptor 2 (TLR2) recognizes and binds to phosphatidylserine oxidized by lipid peroxides (SAPE-OOH) on the surface of ferroptotic cells, enhancing their phagocytic clearance. Additionally, high mobility group box 1 (HMGB1) released by dying cells interacts with the receptor for advanced glycation end products (RAGE) on macrophages, mediating inflammatory responses. Chemokines such as CCL2 and CCL7 further recruit macrophages and promote chemotaxis, amplifying immune reactions. Iron accumulation within macrophages drives M1 polarization through three primary mechanisms: upregulation of M1 markers (e.g., IL-6, TNF-α, IL-1β) and downregulation of M2 markers (e.g., TGM2); enhancement of glycolysis favoring an M1 phenotype; and induction of ROS elevation and p53 acetylation, which collectively promote an M1-like macrophage state.

Signal transducer and activator of transcription 3 (STAT3) is a key transcription factor in the ferroptosis-macrophage polarization interplay. Upon RAGE activation, a cascade of signaling events leads to phosphorylation of STAT3 on tyrosine residues. Phosphorylated STAT3 forms dimers, translocates to the nucleus, and binds specific sequences in target gene promoters to regulate transcription. In macrophage polarization, STAT3 activation is crucial for M2 polarization. STAT3 promotes the expression of M2-associated genes like arginase 1 (Arg1), mannose receptor (CD206), and interleukin-10 (IL-10). The products of these genes participate in M2 functions like immunoregulation and tissue repair. In ferroptosis-induced polarization, activation of the RAGE-STAT3 pathway enhances M2 polarization, affecting the immune state of the tumor microenvironment and tumor progression. Research in pancreatic ductal adenocarcinoma shows that ferroptotic cancer cells release KRAS G12D protein, which can be taken up by macrophages via exosomes, with RAGE playing a key mediating role. After uptake, KRAS G12D promotes fatty acid oxidation and M2 polarization in macrophages via the RAGE-STAT3 pathway, fostering tumor growth and metastasis [132].

Beyond the RAGE-STAT3 pathway, other signaling pathways participate in the interaction. The nuclear factor-kappa B (NF-κB) pathway plays a significant role in ferroptosis-induced M1 polarization. Oxidative stress and inflammatory responses during ferroptosis can activate NF-κB, promoting the expression of M1 genes like iNOS, IL-1β, and TNF-α [133]. The mitogen-activated protein kinase (MAPK) pathway is also closely related to both processes, influencing them by regulating various intracellular biological processes [109]. These pathways interact, forming a complex signaling network that collectively regulates the interplay between ferroptosis and macrophage polarization.

## Role of the ferroptosis-macrophage polarization axis in diseases

### Role in oncological diseases

#### Ferroptosis and macrophage polarization in the tumor microenvironment

The tumor microenvironment (TME) is a complex ecosystem comprising tumor cells, immune cells, stromal cells, and extracellular matrix. Ferroptosis and macrophage polarization play crucial roles within it, and their interaction profoundly influences tumor cell growth, metastasis, and immune escape [134, 135, 136].

**Fig. 5:**
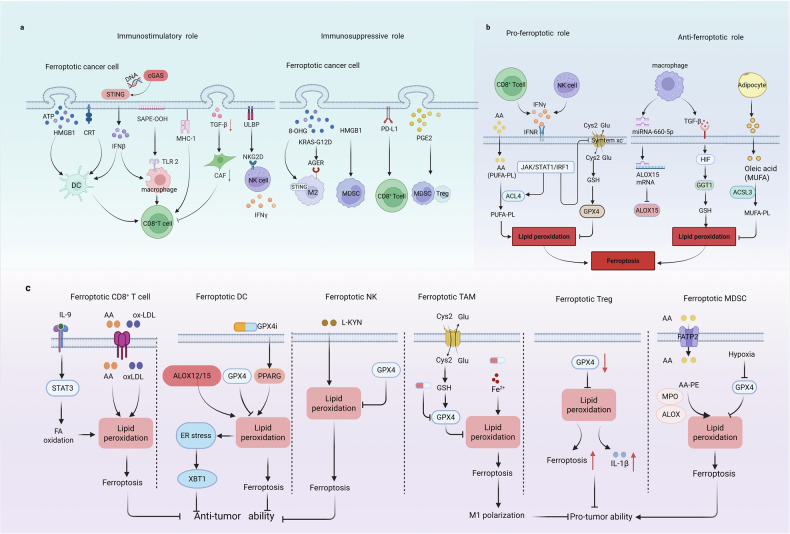
Ferroptosis-mediated crosstalk in the tumor microenvironment (TME). **a** illustrates the dual immunoregulatory role of ferroptotic cancer cells within the tumor microenvironment (TME). These cells can exert both immunostimulatory and immunosuppressive effects through the release of distinct immunomodulatory signals, thereby differentially influencing tumor progression. **b** summarizes the pro-ferroptotic and anti-ferroptotic impacts on cancer cells, which are mediated by other cellular components in the TME, notably immune cells and adipocytes. **c** details the mechanisms and tumor-modulating consequences of ferroptosis occurring within various immune cell populations in the TME, including CD8⁺ T cells, dendritic cells (DCs), natural killer (NK) cells, tumor-associated macrophages (TAMs), regulatory T cells (Tregs), and myeloid-derived suppressor cells (MDSCs).

Within the TME, ferroptosis in tumor cells can influence macrophage polarization through multiple avenues (Fig. 5). When tumor cells undergo ferroptosis, they release substantial amounts of damage-associated molecular patterns (DAMPs), such as high mobility group box 1 (HMGB1) and heat shock proteins (HSPs). These DAMPs can be recognized by pattern recognition receptors (PRRs) on macrophages, activating intracellular signaling and influencing polarization direction. Studies show that HMGB1 can bind to the receptor for advanced glycation end products (RAGE) on macrophages, activating the RAGE-STAT3 pathway and promoting M2 polarization. This shift leads to increased secretion of anti-inflammatory cytokines like IL-10 and TGF-β, suppressing immune responses and creating a favorable condition for tumor growth and metastasis [137]. Lipid peroxidation products generated during tumor cell ferroptosis, such as malondialdehyde (MDA) and 4-hydroxynonenal (4-HNE), can also act as signaling molecules regulating macrophage polarization. These products can activate pathways like MAPK and NF-κB in macrophages, promoting M1 polarization [138]. M1 macrophages possess strong anti-tumor activity, secreting pro-inflammatory cytokines like TNF-α, IL-1β, and IL-6, activating other immune cells, and enhancing anti-tumor immunity [139]. In some cases, lipid peroxidation products from ferroptotic tumor cells might also cause macrophage dysfunction, impairing their anti-tumor efficacy [140].

Furthermore, tumor-associated macrophages (TAMs) are a specific phenotype of M2-like macrophages and represent a major subpopulation of immune cells in the tumor microenvironment (TME) functioning as phagocytes of undetermined origin. TAMs have been extensively studied for their pro-tumor activities, including tumorigenesis, angiogenesis, metastasis, drug resistance, and anti-tumor immunosuppression [141, 142]. Indeed, TAMs are major components of the TME [143], some characteristics of TAMs strongly resemble M2 polarization, such as the high production of IL-10 and TGF-β, which are critically involved in the immune escape and angiogenesis of tumor cells [144, 145]. Within the TME, ferroptosis influences the M2 polarization of TAMs through various mechanisms. Research indicates that TMEM147 induces ferroptosis by promoting the activation of the 27HC/GPX4 pathway in hepatocellular carcinoma cells, which enhances lipid metabolism in macrophages, leading to the metabolic reprogramming of TAMs and driving their M2 polarization [146]. Furthermore, a recent study revealed that oxidative stress induced the release of the KRASG12D protein from cancer cells that succumbed to autophagy-dependent ferroptosis to the TME, which in turn drove the polarization of macrophages in the TME to the M2 phenotype to promote the growth of pancreatic cancer [132].

Conversely, when TAMs themselves undergo ferroptosis, it can remodel the TME to significantly enhance the efficacy of anti-tumor therapies. For instance, M2-like TAMs typically create an anti-ferroptotic microenvironment by highly expressing iron export proteins and heme degradation pathway components (HMOX1), producing the antioxidant glutathione (GSH), and secreting IL-10 and TGF-β. By modulating iron uptake and storage in M2 macrophages while inhibiting iron release, iron accumulation within TAMs can enhance reactive oxygen species (ROS) production, increase p300/CBP acetyltransferase activity, and promote p53 acetylation. These molecular changes drive the transformation towards a pro-inflammatory M1 phenotype possessing anti-tumor properties [147]. Additionally, knocking out xCT in macrophages reduces the expression of phosphorylated STAT6 and PPAR-γ, and enhances SOCS3 expression, thereby inhibiting M2 macrophage polarization [148]. Therefore, selectively inducing ferroptosis in M2 TAMs has emerged as a highly promising therapeutic breakthrough. Triggering ferroptotic death in this specific macrophage population not only directly eliminates the pro-tumor M2 cells but also releases relevant signals to reprogram surviving macrophages or recruit new monocytes to differentiate into the anti-tumor M1 phenotype, thereby reshaping the immune microenvironment and inhibiting tumor progression.

Based on the aforementioned studies, a critical question arises: how does the occurrence of ferroptosis in TAMs affect the metabolic state and the subsequent ferroptosis of adjacent tumor cells? Recent research demonstrates that when macrophages are targeted with ferroptosis inducers, the macrophages and their derived extracellular vesicles can deliver peroxiredoxin 6 (PRDX6) to tumor cells. PRDX6 utilizes its glutathione peroxidase activity to reduce the accumulation of mitochondrial superoxide and inhibit ferroptosis-associated mitophagy. This mechanism effectively compensates for the inhibited function of GPX4, thereby conferring ferroptosis resistance to tumor cells and promoting tumor progression. Concurrently, targeting the macrophages in the TME or directly targeting PRDX6 can reverse this ferroptosis resistance, providing a novel preclinical rationale for tumor therapies based on ferroptosis induction [149].

Changes in macrophage polarization also significantly affect the ferroptosis sensitivity of tumor cells. M1 macrophages secrete various cytokines and ROS that can directly or indirectly induce ferroptosis in tumor cells. TNF-α from M1 cells can activate death receptor pathways in tumor cells, inducing apoptosis, and can also activate the NF-κB pathway, promoting the expression of ferroptosis-related genes like TFR1, increasing tumor cell sensitivity to ferroptosis [150]. ROS produced by M1 macrophages, such as O₂⁻·, H₂O₂, and ·OH, can directly attack PUFAs in tumor cell membranes, initiating lipid peroxidation chain reactions and causing ferroptosis [151]. Conversely, M2 macrophages tend to inhibit ferroptosis in tumor cells. Anti-inflammatory cytokines like IL-10 and TGF-β from M2 cells suppress immune responses and reduce ROS production, lowering oxidative stress in tumor cells and inhibiting ferroptosis [152]. M2 macrophages can also modulate tumor cell iron metabolism, reducing intracellular iron levels and ferroptosis. By highly expressing ferroportin (FPN), M2 macrophages can promote iron export from tumor cells, decreasing intracellular iron concentration and inhibiting ferroptosis [122].

Ferroptosis and macrophage polarization in the TME significantly impact tumor cell growth, metastasis, and immune escape. Ferroptosis can act as a natural anti-cancer mechanism, limiting tumor growth and spread. Some tumor cells are particularly sensitive to ferroptosis due to their metabolic traits, making its induction a potential therapeutic strategy. In the TME, ferroptosis inducers can cause tumor cell death, releasing DAMPs that activate immune responses and enhance immune surveillance and killing. Changes in macrophage polarization also affect tumor growth and metastasis. The anti-tumor activity of M1 macrophages can inhibit tumor growth and metastasis, while M2 macrophages promote tumor growth, angiogenesis, and metastasis. M2 macrophages secrete pro-angiogenic factors like vascular endothelial growth factor (VEGF), promoting tumor vascularization to supply nutrients and oxygen, facilitating growth and metastasis [153]. M2 macrophages also secrete proteinases like matrix metalloproteinases (MMPs), degrading the extracellular matrix and promoting tumor cell invasion and metastasis [154]. The interplay between ferroptosis and macrophage polarization in the TME is also closely linked to tumor immune escape. Anti-inflammatory cytokines like IL-10 and TGF-β from M2 macrophages suppress immune responses, reducing immune surveillance and killing of tumor cells, promoting immune escape [155]. DAMPs released during tumor cell ferroptosis, if not effectively cleared, may also induce immune tolerance, further facilitating immune escape [155].

#### Cancer therapeutic strategies targeting ferroptosis and macrophage polarization

Given the important roles of ferroptosis and macrophage polarization in the TME, developing therapeutic strategies targeting these processes holds significant clinical promise. Several approaches aiming to disrupt the TME and enhance anti-tumor immunity by modulating ferroptosis and macrophage polarization have been proposed.

Application of Ferroptosis Inducers is a key strategy. Using inducers like Erastin and RSL3 can directly trigger ferroptosis in tumor cells, inhibiting growth. Erastin primarily inhibits System Xc − , reducing cystine uptake and GSH synthesis, depriving GPX4 of its substrate. RSL3 directly inhibits GPX4 enzyme activity. Preclinical studies have shown efficacy against various cancer cells. For example, Erastin effectively induces ferroptosis and inhibits proliferation/migration in breast cancer cells [156, 157]. However, potential toxicity to normal cells requires optimization of dosing and delivery for safety and efficacy.

Modulation of Macrophage Polarization is another promising strategy. Promoting M1 polarization can enhance anti-tumor activity. This can be achieved using cytokines, small molecules, or nanomaterials. Cytokines like IFN-γ and LPS can induce M1 polarization. Modulating tumor-associated macrophages (TAMs) represents a highly promising avenue for cancer therapy, In mouse models, administration of IFN-γ and LPS promoted TAM repolarization to M1, enhancing anti-tumor immunity and inhibiting growth [158]. Emerging studies propose that ferroptosis-enhanced macrophage regulation strategies provide robust support for TAM-centric anti-tumor interventions [159]. Small molecules like curcumin and quercetin can modulate polarization. Curcumin can promote M1 polarization by inhibiting NF-κB [160, 161]. Furthermore, conventional treatments such as radiotherapy (RT) not only exert direct cytotoxicity on tumor cells but also profoundly impact the tumor microenvironment (TME), frequently leading to the unintended accumulation of radioresistant M2-like TAMs [162]. Nanomaterials like gold nanoparticles can be taken up by macrophages and influence polarization via intracellular signaling, e.g., by activating TLR4 to promote M1 polarization [163, 164].

Combination Therapies are a current research focus. Combining ferroptosis inducers with macrophage polarization modulators can yield synergistic effects. For instance, combining a ferroptosis inducer (RSL3) with IFN-γ in a mouse model showed enhanced tumor growth inhibition and prolonged survival, indicating synergistic anti-tumor effects [165, 166]. Combining ferroptosis inducers with immune checkpoint inhibitors (e.g., PD-1 inhibitors) is another promising approach. Checkpoint inhibitors relieve tumor-induced immunosuppression, while ferroptosis inducers induce immunogenic cell death, releasing antigens. Their combination in a melanoma mouse model enhanced T cell infiltration and anti-tumor immunity [167].

Building on these foundations, recent breakthroughs in advanced materials have provided highly innovative, targeted tools for remodeling the tumor microenvironment (TME). For example, a novel carrier-free nanomedicine was recently developed. This stable, self-assembling nanoparticle (BC NPs) was constructed from the photosensitizer chlorin e6 (Ce6) and baicalin, a natural product isolated from Scutellaria baicalensis, utilizing π-π stacking, hydrogen bonding, and hydrophobic interactions. Baicalin enhances the efficacy of photodynamic therapy by depleting glutathione (GSH) levels in tumors, thereby triggering programmed ferroptosis in tumor cells. Concurrently, baicalin promotes the polarization of M1 macrophages in the TME, leading to TNF-α production and exerting anti-tumor effects. This synergizes with the immunogenic cell death induced by photodynamic therapy to effectively reprogram immunosuppressive M2 macrophages into the tumoricidal M1 phenotype. Consequently, this approach alleviates TME immunosuppression and induces a durable and robust anti-tumor immune response [168]. Similarly, in the realm of radioimmunotherapy, advanced organic aggregation-induced emission (AIE) nanoradiosensitizers have been engineered to trigger continuous reactive oxygen species (ROS) generation under X-ray irradiation. This sustained ROS burst not only induces severe oxidative stress and subsequent ferroptotic damage in tumor cells but also plays a crucial role in remodeling the immune microenvironment, fostering a robust pro-inflammatory macrophage response that potentiates systemic anti-tumor immunity [169]. These cutting-edge nanomedicine strategies highlight the immense therapeutic potential of coupling ROS/ferroptosis induction with precise macrophage repolarization [170].

### Role in inflammatory diseases

#### Alterations in ferroptosis and macrophage polarization in the inflammatory microenvironment

The inflammatory microenvironment is a complex biological setting formed by a dynamic network of immune cells, stromal cells, cytokines, and metabolites. During inflammation, a “bidirectional regulatory axis” is established, where molecular cross-talk determines the initiation, amplification, or resolution of inflammation.

**Fig. 6:**
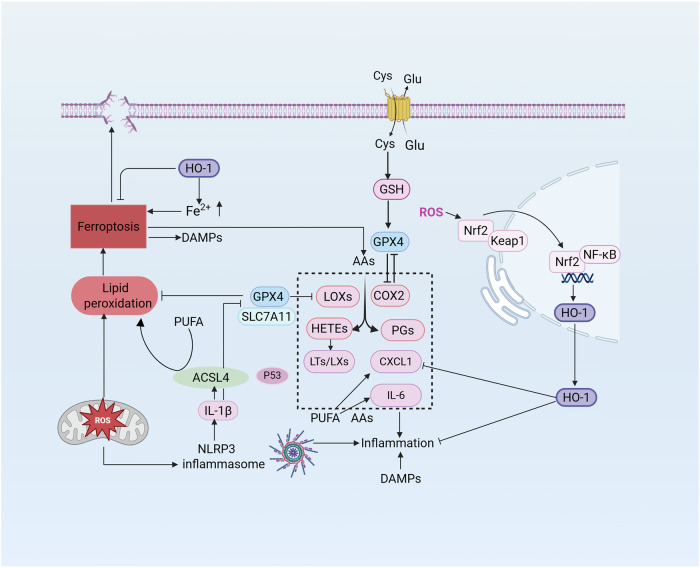
The link between ferroptosis and inflammation. Ferroptosis is closely related to inflammation. Through the mediums of some ferroptosis regulators, such as GPX4, ROS, LOXs, and inflammatory mediators produced in the process of ferroptosis, ferroptosis can aggravate inflammatory response to varying degrees.

Ferroptosis is closely related to inflammation. Through the mediums of some ferroptosis regulators, such as GPX4, ROS, LOXs, and inflammatory mediators produced in the process of ferroptosis, ferroptosis can aggravate inflammatory response to varying degrees (Fig. 6).

When the body is challenged by pathogens or tissue injury, an inflammatory response ensues, creating an inflammatory microenvironment. Within it, concentrations of bioactive substances like cytokines, chemokines, and ROS change, influencing ferroptosis and macrophage polarization. Inflammatory cytokines can regulate ferroptosis-related gene expression, affecting cellular sensitivity. TNF-α, a key pro-inflammatory cytokine elevated in inflammation, can activate RIPK1/RIPK3/MLKL-dependent necroptosis, a process closely related to ferroptosis [127, 171]. TNF-α can also activate NF-κB, promoting expression of ferroptosis-related genes like TFR1, increasing sensitivity [172]. IL-1β, another common inflammatory cytokine, can activate the NLRP3 inflammasome, promoting cytokine release and inflammation [173]. NLRP3 activation leads to caspase-1 activation, processing pro-IL-1β. IL-1β can act on neighboring cells, regulating their iron metabolism and oxidative stress, influencing ferroptosis [174].

NLRP3 activation leads to caspase-1 activation, processing pro-IL-1β. IL-1β can act on neighboring cells, regulating their iron metabolism and oxidative stress, influencing ferroptosis [173].

Macrophage polarization undergoes significant changes in the inflammatory microenvironment. Macrophages can polarize to M1 or M2 phenotypes, impacting inflammation initiation, progression, and resolution. In early inflammation, M1 polarization dominates, playing a pro-inflammatory role. M1 macrophages secrete large amounts of TNF-α, IL-1β, IL-6, and IL-12, activating other immune cells and enhancing inflammation to clear pathogens. They also produce copious NO for cytotoxicity. As inflammation progresses, macrophages gradually shift towards M2 polarization. M2 macrophages primarily exert anti-inflammatory and tissue-repair functions in later stages, secreting cytokines like IL-10 and TGF-β. IL-10 inhibits other immune cells, reducing pro-inflammatory cytokine production. TGF-β promotes fibroblast proliferation and collagen synthesis, aiding repair and fibrosis.

Ferroptosis and macrophage polarization interact within the inflammatory milieu, co-regulating inflammation. Ferroptosis can influence macrophage polarization by releasing DAMPs. Cells undergoing ferroptosis release HMGB1, ATP, etc., which are recognized by PRRs on macrophages, activating signaling and affecting polarization direction. HMGB1 binding to RAGE can activate the RAGE-STAT3 pathway, promoting M2 polarization [132]. Conversely, changes in macrophage polarization affect ferroptosis. M1-derived cytokines and ROS promote ferroptosis, while M2-derived anti-inflammatory cytokines inhibit it. M1-secreted TNF-α can promote ferroptosis sensitivity via NF-κB. M2-secreted IL-10 can inhibit immune responses and ROS production, reducing oxidative stress and inhibiting ferroptosis.

#### Potential therapeutic targets for related diseases

Given the important roles and interplay of ferroptosis and macrophage polarization in inflammation, targeting their connection holds broad prospects for developing therapies for inflammatory diseases.

Modulating Ferroptosis-Related Pathways is a potential target (Table 1). Regulating iron metabolism proteins can influence ferroptosis. Inhibiting transferrin receptor 1 (TFR1) expression/activity can reduce cellular iron uptake, lowering intracellular iron and inhibiting ferroptosis. In inflammatory bowel disease, inhibiting TFR1 alleviated intestinal epithelial cell ferroptosis and inflammation [112]. Promoting ferritin expression or enhancing its iron storage capacity can reduce labile iron pool Fe²⁺ levels, inhibiting ferroptosis. Some studies show that upregulating ferritin expression via gene editing or drugs can alleviate inflammation-related tissue damage [175, 176]. Modulating glutathione peroxidase 4 (GPX4) activity is another approach. Enhancing GPX4 activity can inhibit lipid peroxidation, preventing ferroptosis. Small molecule GPX4 agonists could be used to reduce tissue injury in inflammatory diseases [177]. Conversely, in contexts like cancer, inducing ferroptosis using GPX4 inhibitors like RSL3 might be desirable [36, 177, 178].

**Table 1:** A Systematic Overview of Therapeutic Targets and Pharmacological Agents in Ferroptosis.

Target	Mechanism	Representative Drugs/Molecules	Primary Direction	Effect
System xc-	Inhibits cyctine import, depleting gluathione	Sulfasalazine(SASP), Erastin, Sorafenib	Cancer Therapy	Induce
	Lanperisone-mediated induction of intracellular reactive oxygen species	Lanperisone	K-ras mutant tumors	Induce
GPX4	Directly inhibits GPX4 enzyme activity	RSL3, ML162, FINO₂, Altretamine	Cancer Therapy	Induce
GSH Synthesis	Limits precursors for glutathione synthesis	CB-839 (glutaminase inhibitor)	Cancer Therapy	Induce (Sensitizer)
FSP1	Inhibits the CoQH2 regeneration system on the plasma membrane	iFSP1	Cancer Therapy	Induce (Sensitizer)
DHODH	Inhibits the mitochondrial CoQH2 regeneration system	Brequinar	Cancer Therapy	Induce
**Iron Metabolism**	Chelates free iron, inhibiting the Fenton reaction	Artesunate, Iron-based nanoparticles	Cytoprotection	Induce
	Artesunate, Iron-based nanoparticles	Deferoxamine (DFO), Deferiprone	Cytoprotection	Inhibit
Lipid Peroxidation	Traps lipid radicals	Ferrostatin-1, Liproxstatin-1, Vitamin E	Cytoprotection	Inhibit
**Selenium**	Acts as a cofactor for the synthesis of functional GPX4	Sodium Selenite	Cytoprotection/Sensitization	Inhibit (or bidirectional)
**Mevalonate Pathway**	Inhibits CoQ10 synthesis, weakening the FSP1/DHODH pathways	Statins (e.g., Simvastatin)	Cancer Therapy	Sensitizer
**p53 Pathway**	Transcriptionally represses SLC7A11	APR-246 (p53 activator)	Cancer Therapy	Induce (Indirect)
**Hippo-YAP**	Upregulates pro-ferroptosis genes such as ACSL4 and TFRC	Potential target; under drug development.	Cancer Therapy	Sensitizer

*ACSL4* Acyl-CoA synthetase long-chain family member 4, *CoQ10/CoQH2* Coenzyme Q10 (oxidized/reduced form), *DHODH* Dihydroorotate dehydrogenase, *FSP1* Ferroptosis suppressor protein 1, *GPX4* Glutathione peroxidase 4, *GSH* Glutathione, *LOX* Lipoxygenase, *SASP* Sulfasalazine, *System Xc*- Cystine/glutamate antiporter (SLC7A11/SLC3A2 complex), *TFRC* Transferrin receptor.

Regulating Macrophage Polarization is another key strategy. Modulating polarization-related signaling pathways and transcription factors can shift macrophage phenotypes. Inhibiting the NF-κB pathway, central to M1 polarization, can reduce M1 polarization and pro-inflammatory cytokine secretion, alleviating inflammation. Natural products like curcumin and quercetin can inhibit NF-κB [169, 179, 180]. Activating the STAT6 pathway, key for M2 polarization, can promote M2 polarization and enhance anti-inflammatory/repair capacity [85, 181]. Cytokines like IL-4 and IL-13 can be used to activate STAT6. Other transcription factors like IRF5 and PPAR-γ can also be targeted to influence polarization.

Combined Targeting of Ferroptosis and Macrophage Polarization may yield better outcomes. In inflammatory diseases, the two processes interact. Simultaneous modulation could more comprehensively control inflammation and promote repair. Combining a ferroptosis inducer with an M2-promoting agent is one strategy: the inducer clears damaged/infected cells and releases DAMPs, followed by an agent like IL-4 to promote M2 polarization for anti-inflammatory and reparative effects. Alternatively, combining a ferroptosis inhibitor with an M1 inhibitor could be used. In inflammatory bowel disease, a GPX4 agonist could inhibit epithelial ferroptosis, while an NF-κB inhibitor could suppress M1 polarization, reducing pro-inflammatory cytokines and alleviating symptoms.

### Role in other diseases

Beyond cancer and inflammatory diseases, the interplay between ferroptosis and macrophage polarization shows significant research progress and potential implications in cardiovascular and neurodegenerative diseases, among others.

#### Role in cardiovascular diseases

In cardiovascular diseases, this interplay is particularly prominent in atherosclerosis (AS). The core pathological feature of AS is atherosclerotic plaque formation, where macrophages are key cells within the plaque microenvironment. After macrophages uptake oxidized low-density lipoprotein (ox-LDL) and transform into foam cells, their polarization state closely relates to ferroptosis sensitivity. M1 macrophages can exacerbate local oxidative stress by releasing pro-inflammatory factors (e.g., TNF-α, IL-1β), activating ferroptosis pathways and increasing foam cell ferroptosis. The free iron and lipid peroxides released from dying cells further recruit more macrophages and promote their M1 polarization, forming a vicious “inflammation-ferroptosis” cycle that accelerates plaque necrotic core expansion and instability. Conversely, M2 macrophages can inhibit oxidative stress by secreting anti-inflammatory factors like IL-10, while upregulating ferroptosis inhibitory proteins (e.g., GPX4, SLC7A11), reducing foam cell ferroptosis and helping stabilize plaques. Studies indicate that erythrophagocytosis-induced ferroptosis during intraplaque angiogenesis promotes plaque enlargement, an effect preventable by the ferroptosis inhibitor UAMC-3203 [182]. Smoking has also been reported to induce macrophage ferroptosis via the NF-κB-activated transferrin/FPN/SLC7A11 pathway, promoting AS progression [109]. Additionally, alleviating lipid peroxidation to inhibit ferroptosis can mitigate atherosclerosis [183]. In myocardial infarction (MI) and ischemia-reperfusion injury, the interaction influences myocardial repair. Early post-infarction, the ischemic/hypoxic environment induces cardiomyocyte ferroptosis. Released DAMPs recruit macrophages and promote M1 polarization. M1 macrophages, by enhancing iron accumulation and lipid peroxidation, further expand cardiomyocyte ferroptosis, aggravating inflammatory injury. During the repair phase, M2 macrophages become dominant. Their secreted TGF-β and other cytokines can inhibit ferroptosis pathways while promoting fibroblast proliferation and angiogenesis, reducing myocardial fibrosis. Research shows that regulating macrophage shift from M1 to M2 significantly reduces cardiomyocyte ferroptosis and improves post-infarction cardiac function [184]. In heart failure, chronic inflammation and oxidative stress sustain a dominant M1 state, accelerating cardiomyocyte ferroptosis, reducing cardiomyocyte numbers, and impairing contractility. Promoting M2 polarization may inhibit ferroptosis and enhance cardiomyocyte survival, delaying heart failure progression

#### Role in neurodegenerative diseases

In neurodegenerative diseases, the interaction between ferroptosis and macrophage (microglial) polarization is also a key for understanding disease mechanisms, showing unique and important regulatory roles in Alzheimer’s disease (AD) and Parkinson’s disease (PD).

Alzheimer’s Disease (AD): Core pathological features include β-amyloid (Aβ) plaques and tau neurofibrillary tangles, with brain iron dyshomeostasis, chronic neuroinflammation, and neuronal death being closely linked. Microglia, the resident immune cells of the CNS, can be activated into pro-inflammatory M1 or anti-inflammatory M2 phenotypes, influencing ferroptosis. Aβ deposition can activate microglia towards M1. M1 microglia release pro-inflammatory cytokines like IL-1β and TNF-α, exacerbating brain oxidative stress, while upregulating transferrin receptor (TfR1) expression, promoting neuronal iron accumulation. Additionally, M1 cells inhibit glutathione peroxidase 4 (GPX4) activity, weakening neuronal capacity to clear lipid peroxides, ultimately triggering neuronal ferroptosis. Studies confirm increased M1 microglia around plaques in AD brains, with neuronal iron content and lipid peroxidation products (e.g., 4-HNE) positively correlated in these regions [185]. M2 microglia can inhibit neuroinflammation by secreting IL-10 and TGF-β, while upregulating iron exporter ferroportin (FPN), promoting excess iron efflux. Moreover, M2 microglia can enhance GPX4 and solute carrier family 7 member 11 (SLC7A11, a key glutathione synthesis transporter) expression, maintaining neuronal glutathione levels and inhibiting ferroptosis. Experiments show that drug-induced M2 polarization significantly reduces neuronal ferroptosis, lowers Aβ deposition, and improves cognition in AD mouse models [185]. Studies have shown that NOX4 promotes astrocyte ferroptosis in Alzheimer’s disease (AD) through oxidative stress-induced lipid peroxidation and impaired mitochondrial metabolism, serving as an important molecular mechanism [186].

Parkinson’s disease (PD) is primarily characterized by the progressive loss of dopaminergic (DA) neurons in the substantia nigra pars compacta. Key pathogenic factors include abnormal brain iron accumulation, α-synuclein (α-syn) aggregation, and neuroinflammation. The interplay between ferroptosis and microglial polarization plays a crucial regulatory role in this process. Pathologically aggregated α-syn can activate the toll-like receptor 4 (TLR4)/NF-κB signaling pathway, inducing microglial polarization towards the M1 phenotype. M1 microglia exacerbate oxidative stress in the substantia nigra by releasing reactive oxygen species (ROS) and pro-inflammatory factors. Simultaneously, they downregulate the expression of ferroportin (FPN), leading to massive iron accumulation within DA neurons. DA neurons themselves are rich in dopamine, whose metabolism easily generates quinones, further promoting lipid peroxidation. This ultimately activates the ferroptosis pathway, resulting in DA neuronal death. Research has found that the number of M1 microglia in the substantia nigra of PD patients positively correlates with iron content, and the expression levels of ferroptosis markers (e.g., ACSL4, PTGS2) are significantly higher than in healthy individuals [187]. M2 microglia can phagocytose and clear abnormally aggregated α-syn, reducing the pathological stimulus source. They also secrete brain-derived neurotrophic factor (BDNF) and glial cell line-derived neurotrophic factor (GDNF), enhancing the resilience of DA neurons against damage. Furthermore, M2 cells can upregulate the expression of GPX4 and SLC7A11, inhibiting the activation of ferroptosis pathways. Animal experiments show that transplanting M2 microglia into the brains of PD model rats significantly reduces the loss of DA neurons in the substantia nigra and improves motor dysfunction [122, 188].

#### Potential therapeutic targets for related diseases

Given the core regulatory roles of the ferroptosis-macrophage/microglia polarization axis in cardiovascular and neurodegenerative diseases, the key molecules and signaling pathways within their interaction mechanism have become important potential therapeutic targets.

Intervention in Key ferroptosis pathways is a primary therapeutic target. These targets act directly on the core “iron accumulation-lipid peroxidation-antioxidant system” of ferroptosis, blocking the process at the molecular level. Research has demonstrated that Thonningianin A (ThA), a novel ferroptosis inhibitor, significantly improves AD-related pathological changes by counteracting neuronal ferroptosis. This effect is achieved through its interaction with GPX4 and activation of the AMPK/Nrf2/GPX4 signaling pathway [186].

It has been confirmed that SZRD (SuanZaoRen decoction) activates the DJ-1/Nrf2 signaling pathway. It upregulates protein expression levels of FPN1, DJ-1, Nrf2, GPX4, and SLC7A11 in the hippocampal region while inhibiting the expression of TfR1, FTH1, FTL, and ACSL4 proteins, suppressing Aβ deposition. Consequently, it alleviates neuronal loss, hippocampal synaptic damage, and ferroptosis in APP/PS1 mice [189].

As the microenvironment mediated by microglial (MG) M1/M2 phenotypic switching plays a decisive role in neuronal state and cognitive function in Alzheimer’s disease (AD), regulation of macrophage/microglia polarization is another key therapeutic target. Research reveals that cordycepin targets hexokinase II (HKII) to elevate the extracellular acidification rate (ECAR) in the glycolytic pathway, while targeting pyruvate dehydrogenase kinase 2 (PDK2) to enhance the oxygen consumption rate (OCR) mediated by the pyruvate dehydrogenase (PDH)-driven oxidative phosphorylation (OXPHOS) pathway. This induces MG-M2 polarization, improves cognitive function and memory in APP/PS1 mice, promotes neuronal survival, and exerts anti-AD effects [190]. Research results indicate that Pterosin B (PB) improves cognitive dysfunction in AD by regulating microglial M1/M2 polarization through inhibiting the Klf5/Parp14 pathway [191].

Since the interaction between ferroptosis and macrophage/microglia polarization involves multiple pathways and molecules, single-target therapy may have limited efficacy. Combination therapeutic strategies are expected to be a major breakthrough in treating neurodegenerative diseases. Studies show that magnolol alleviates depressive-like behavior by polarizing microglia to an M2 phenotype via regulating the Nrf2/HO-1/NLRP3 signaling pathway [192]. M1 macrophages/microglia are involved in neuronal pyroptosis and have recently been reported as an anti-ferroptotic phenotype. Research finds that quercetin inhibits the extent of neuronal pyroptosis and ferroptosis in atherosclerosis by modulating microglial M1/M2 polarization, thereby alleviating neuroinflammation and protecting neurons from ferroptosis. This may provide a novel direction for the prevention and treatment of neuroinflammation [193].

## Conclusion and perspectives

### Research summary

This study has thoroughly investigated the interaction mechanisms between ferroptosis and macrophage polarization and their roles in diseases, achieving a series of significant results. Ferroptosis, as a novel form of regulated cell death, provides a new perspective for understanding cell fate regulation through its unique biological mechanisms. Research on the molecular regulatory mechanisms of ferroptosis has clarified the central role of GPX4-related pathways in ferroptosis regulation, as well as the important influence of disrupted iron metabolism and other related mechanisms on the initiation and progression of ferroptosis. Macrophage polarization is a key process in immune system regulation, with different polarized types playing distinct roles in immune responses, inflammatory reactions, and tissue repair. Investigation into the molecular regulatory mechanisms of macrophage polarization has revealed the crucial regulatory functions of signaling pathways and transcription factors.

More importantly, this study has uncovered the existence of complex and intimate interactions between ferroptosis and macrophage polarization. Ferroptosis can influence the direction of macrophage polarization by inducing changes in macrophage iron metabolism and other metabolic reprogramming events. Conversely, alterations in macrophage polarization states significantly impact the occurrence and development of ferroptosis. M1 macrophages can induce ferroptosis through secreting cytokines, producing ROS and NO, while M2 macrophages can inhibit ferroptosis by regulating iron metabolism and antioxidant capacity. The molecular mechanisms of this interaction involve multiple signaling pathways, such as AGER-STAT3, which are intertwined and collectively regulate the dynamic balance between ferroptosis and macrophage polarization.

In terms of disease research, the association between ferroptosis and macrophage polarization has been found to play important roles in various diseases, including cancer and inflammatory disorders. Within the tumor microenvironment, ferroptosis and macrophage polarization mutually influence each other and jointly act on tumor cell growth, metastasis, and immune escape. By targeting ferroptosis and macrophage polarization, several promising cancer treatment strategies have been developed, offering new ideas and methods for cancer therapy. In inflammatory diseases, changes in ferroptosis and macrophage polarization within the inflammatory microenvironment interact, affecting the initiation, development, and resolution of inflammation. Targeting their association provides potential directions for drug development and intervention measures in treating inflammatory diseases.

The findings of this study not only enrich the understanding of the biological mechanisms of ferroptosis and macrophage polarization but also provide a novel perspective for gaining deeper insights into the pathogenesis of various diseases. They laid a solid theoretical foundation for developing disease treatment strategies based on the regulation of ferroptosis and macrophage polarization, holding significant theoretical importance and potential clinical application value.

### Limitations and future perspectives

Despite advances in understanding the interplay between ferroptosis and macrophage polarization in disease contexts, several limitations remain in current research.

Regarding molecular mechanisms, while some key signaling pathways and regulatory factors have been revealed, the molecular mechanisms of the interaction between ferroptosis and macrophage polarization are not yet fully elucidated. The specific regulatory mechanisms of pathways like AGER-STAT3 in different disease contexts and cell types require further in-depth research. Other key molecules and signaling pathways that have not yet been discovered may also be involved. The interactions and network regulatory relationships between different signaling pathways also need further clarification to comprehensively unveil the molecular basis of the ferroptosis-macrophage polarization interaction.

In terms of clinical application, current research is primarily focused on the stages of cell experiments and animal models. Translating relevant research findings into clinical treatment methods still faces many challenges. The safety and efficacy of ferroptosis inducers and drugs that modulate macrophage polarization in clinical trials need further verification. How to optimize drug design and administration regimens to improve therapeutic effects and reduce adverse reactions is an urgent problem to be solved. The pathogenesis of complex diseases such as cancer and inflammatory diseases involves the interaction of multiple factors. Targeting ferroptosis or macrophage polarization alone may not achieve ideal therapeutic outcomes. How to develop combination therapy strategies, comprehensively consider multiple factors, and achieve personalized treatment is also a direction that future research needs to focus on.

Looking ahead, in-depth research into the molecular mechanisms of the interaction between ferroptosis and macrophage polarization remains an important research direction in this field. Utilizing advanced technologies such as single-cell sequencing and spatial transcriptomics to deeply study the dynamic changes and interaction mechanisms of ferroptosis and macrophage polarization in different cell types and microenvironments is expected to discover new key molecules and signaling pathways, providing more targets for disease treatment. Strengthening the translation from preclinical research to clinical trials, closely collaborating with clinicians, and conducting multicenter, large-sample clinical trials to verify the safety and efficacy of related therapeutic strategies will promote the clinical application of research. Integrating artificial intelligence and big data analysis technologies for precise prediction and assessment of disease pathogenesis, therapeutic effects, and prognosis can provide a scientific basis for personalized treatment. Future research can also expand into other related fields such as developmental biology and neuroscience, exploring the role of the ferroptosis-macrophage polarization association in these areas, further enriching the understanding of this biological phenomenon, and providing new ideas and methods for solving more medical and biological problems.

## References

[1] Xie Y, Hou W, Song X, Yu Y, Huang J, Sun X, et al. Ferroptosis: process and function. Cell Death Differ. 2016;23:369–79. 26794443 10.1038/cdd.2015.158 PMC5072448

[2] Dixon SJ, Lemberg KM, Lamprecht MR, Skouta R, Zaitsev EM, Gleason CE, et al. Ferroptosis: an iron-dependent form of nonapoptotic cell death. Cell. 2012;149:1060–72. 22632970 10.1016/j.cell.2012.03.042 PMC3367386

[3] Li J, Cao F, Yin H, Huang Z, Lin Z, Mao N, et al. Ferroptosis: past, present and future. Cell Death Dis. 2020;11:88. 32015325 10.1038/s41419-020-2298-2 PMC6997353

[4] Doll S, Freitas FP, Shah R, Aldrovandi M, Da Silva MC, Ingold I, et al. Fsp1 is a glutathione-independent ferroptosis suppressor. Nature. 2019;575:693–8. 31634899 10.1038/s41586-019-1707-0

[5] Doll S, Proneth B, Tyurina YY, Panzilius E, Kobayashi S, Ingold I, et al. Acsl4 dictates ferroptosis sensitivity by shaping cellular lipid composition. Nat Chem Biol. 2017;13:91–8. 27842070 10.1038/nchembio.2239 PMC5610546

[6] Zou Y, Palte MJ, Deik AA, Li H, Eaton JK, Wang W, et al. A gpx4-dependent cancer cell state underlies the clear-cell morphology and confers sensitivity to ferroptosis. Nat Commun. 2019;10:1617. 30962421 10.1038/s41467-019-09277-9 PMC6453886

[7] Liang D, Minikes AM, Jiang X. Ferroptosis at the intersection of lipid metabolism and cellular signaling. Mol Cell. 2022;82:2215–27. 35390277 10.1016/j.molcel.2022.03.022 PMC9233073

[8] Sica A, Mantovani A. Macrophage plasticity and polarization: in vivo veritas. J Clin Investig. 2012;122:787–95. 22378047 10.1172/JCI59643 PMC3287223

[9] Mosser DM, Edwards JP. Exploring the full spectrum of macrophage activation. Nat Rev Immunol. 2008;8:958–69. 19029990 10.1038/nri2448 PMC2724991

[10] Mantovani A, Sica A, Sozzani S, Allavena P, Vecchi A, Locati M. The chemokine system in diverse forms of macrophage activation and polarization. Trends Immunol. 2004;25:677–86. 15530839 10.1016/j.it.2004.09.015

[11] Viola A, Munari F, Sánchez-Rodríguez R, Scolaro T, Castegna A. The metabolic signature of macrophage responses. Front Immunol. 2019;10:1462. 31333642 10.3389/fimmu.2019.01462 PMC6618143

[12] Zhang Z, Zhang F, Guo X, An P, Tao Y, Wang F. Ferroportin1 in hepatocytes and macrophages is required for the efficient mobilization of body iron stores in mice. Hepatology. 2012;56:961–71. 22473803 10.1002/hep.25746

[13] Lu K, Dong S, Xia T, Mao L. Kupffer cells degrade (14)C-labeled few-layer graphene to (14)co(2) in liver through erythrophagocytosis. ACS Nano. 2021;15:396–409. 33150787 10.1021/acsnano.0c07452

[14] Wen Q, Liu J, Kang R, Zhou B, Tang D. The release and activity of HMGB1 in ferroptosis. Biochem Biophys Res Commun. 2019;510:278–83. 30686534 10.1016/j.bbrc.2019.01.090

[15] Keuters MH, Keksa-Goldsteine V, Dhungana H, Huuskonen MT, Pomeshchik Y, Savchenko E, et al. An arylthiazyne derivative is a potent inhibitor of lipid peroxidation and ferroptosis, providing neuroprotection in vitro and in vivo. Sci Rep. 2021;11:3518. 33568697 10.1038/s41598-021-81741-3 PMC7876050

[16] Hsieh C, Hsieh H, Shih F, Wang P, Yang L, Shieh D, et al. An innovative Nrf2 nano-modulator induces lung cancer ferroptosis and elicits an immunostimulatory tumor microenvironment. Theranostics. 2021;11:7072–91. 34093872 10.7150/thno.57803 PMC8171079

[17] Sarode P, Zheng X, Giotopoulou GA, Weigert A, Kuenne C, Günther S, et al. Reprogramming of tumor-associated macrophages by targeting β-catenin/fosl2/arid5a signaling: a potential treatment of lung cancer. Sci Adv. 2020;6:eaaz6105. 32548260 10.1126/sciadv.aaz6105 PMC7274802

[18] Cao Y, Li Y, He C, Yan F, Li J, Xu H, et al. Selective ferroptosis inhibitor liproxstatin-1 attenuates neurological deficits and neuroinflammation after subarachnoid hemorrhage. Neurosci Bull. 2021;37:535–49. 33421025 10.1007/s12264-020-00620-5 PMC8055759

[19] Yang WS, Sriramaratnam R, Welsch ME, Shimada K, Skouta R, Viswanathan VS, et al. Regulation of ferroptotic cancer cell death by GPX4. Cell. 2014;156:317–31. 24439385 10.1016/j.cell.2013.12.010 PMC4076414

[20] Shimada K, Skouta R, Kaplan A, Yang WS, Hayano M, Dixon SJ, et al. Global survey of cell death mechanisms reveals metabolic regulation of ferroptosis. Nat Chem Biol. 2016;12:497–503. 27159577 10.1038/nchembio.2079 PMC4920070

[21] Bersuker K, Hendricks JM, Li Z, Magtanong L, Ford B, Tang PH, et al. The coq oxidoreductase fsp1 acts in parallel to gpx4 to inhibit ferroptosis. Nature. 2019;575:688–92. 31634900 10.1038/s41586-019-1705-2 PMC6883167

[22] Bao W, Zhou X, Zhou L, Wang F, Yin X, Lu Y, et al. Targeting mir-124/ferroportin signaling ameliorated neuronal cell death through inhibiting apoptosis and ferroptosis in the aged intracerebral hemorrhage murine model. Aging Cell. 2020;19:e13235. 33068460 10.1111/acel.13235 PMC7681046

[23] Chen Y, Lee D, Kwan KK, Wu M, Wang G, Zhang MS, et al. Mevalonate pathway promotes liver cancer by suppressing ferroptosis through Coq10 production and selenocysteine-tRNA modification. J Hepatol 2025;83:1338–52. 10.1016/j.jhep.2025.06.034 40653112

[24] Warner GJ, Berry MJ, Moustafa ME, Carlson BA, Hatfield DL, Faust JR. Inhibition of selenoprotein synthesis by selenocysteine tRNA [ser]sec lacking isopentenyladenosine. J Biol Chem. 2000;275:28110–9. 10821829 10.1074/jbc.M001280200

[25] Lee J, Nam M, Son HY, Hyun K, Jang SY, Kim JW, et al. Polyunsaturated fatty acid biosynthesis pathway determines ferroptosis sensitivity in gastric cancer. Proc Natl Acad Sci USA. 2020;117:32433–42. 33288688 10.1073/pnas.2006828117 PMC7768719

[26] Majno G, Joris I. Apoptosis, oncosis, and necrosis. An overview of cell death. Am J Pathol. 1995;146:3–15. 7856735 PMC1870771

[27] Glick D, Barth S, Macleod KF. Autophagy: cellular and molecular mechanisms. J Pathol. 2010;221:3–12. 20225336 10.1002/path.2697 PMC2990190

[28] Parzych KR, Klionsky DJ. An overview of autophagy: morphology, mechanism, and regulation. Antioxid Redox Signal. 2014;20:460–73. 23725295 10.1089/ars.2013.5371 PMC3894687

[29] Rochette L, Dogon G, Rigal E, Zeller M, Cottin Y, Vergely C. Lipid peroxidation and iron metabolism: two cornerstones in the homeostasis control of ferroptosis. Int J Mol Sci 2022;24:449. 10.3390/ijms24010449 PMC9820499 36613888

[30] Frazer DM, Anderson GJ. The regulation of iron transport. Biofactors. 2014;40:206–14. 24132807 10.1002/biof.1148

[31] Sun X, Ou Z, Xie M, Kang R, Fan Y, Niu X, et al. Hspb1 as a novel regulator of ferroptotic cancer cell death. Oncogene. 2015;34:5617–25. 25728673 10.1038/onc.2015.32 PMC4640181

[32] Chen X, Yu C, Kang R, Tang D. Iron metabolism in ferroptosis. Front Cell Dev Biol. 2020;8:590226. 33117818 10.3389/fcell.2020.590226 PMC7575751

[33] Zhang J, Zhang Y, Herman B. Caspases, apoptosis and aging. Ageing Res Rev. 2003;2:357–66. 14522240 10.1016/s1568-1637(03)00026-6

[34] Liu S, Yao S, Yang H, Liu S, Wang Y. Autophagy: regulator of cell death. Cell Death Dis. 2023;14:648. 37794028 10.1038/s41419-023-06154-8 PMC10551038

[35] Ursini F, Maiorino M. Lipid peroxidation and ferroptosis: the role of GSH and gpx4. Free Radic Biol Med. 2020;152:175–85. 32165281 10.1016/j.freeradbiomed.2020.02.027

[36] Yagoda N, von Rechenberg M, Zaganjor E, Bauer AJ, Yang WS, Fridman DJ, et al. Ras-raf-mek-dependent oxidative cell death involving voltage-dependent anion channels. Nature. 2007;447:864–8. 17568748 10.1038/nature05859 PMC3047570

[37] Fang X, Cai Z, Wang H, Han D, Cheng Q, Zhang P, et al. Loss of cardiac ferritin h facilitates cardiomyopathy via slc7a11-mediated ferroptosis. Circ Res. 2020;127:486–501. 32349646 10.1161/CIRCRESAHA.120.316509

[38] Else PL, Kraffe E. Docosahexaenoic and arachidonic acid peroxidation: it’s a within-molecule cascade. Biochim Biophys Acta. 2015;1848:417–21. 25450347 10.1016/j.bbamem.2014.10.039

[39] Pandey AV, Sproll P. Pharmacogenomics of human p450 oxidoreductase. Front Pharmacol. 2014;5:103. 24847272 10.3389/fphar.2014.00103 PMC4023047

[40] Yan B, Ai Y, Sun Q, Ma Y, Cao Y, Wang J, et al. Membrane damage during ferroptosis is caused by oxidation of phospholipids catalyzed by the oxidoreductases por and cyb5r1. Mol Cell. 2021;81:355–69. 33321093 10.1016/j.molcel.2020.11.024

[41] Rochette L, Lorin J, Zeller M, Guilland J, Lorgis L, Cottin Y, et al. Nitric oxide synthase inhibition and oxidative stress in cardiovascular diseases: possible therapeutic targets? Pharmacol Ther. 2013;140:239–57. 23859953 10.1016/j.pharmthera.2013.07.004

[42] Yang WS, Kim KJ, Gaschler MM, Patel M, Shchepinov MS, Stockwell BR. Peroxidation of polyunsaturated fatty acids by lipoxygenases drives ferroptosis. Proc Natl Acad Sci USA. 2016;113:E4966–75. 27506793 10.1073/pnas.1603244113 PMC5003261

[43] Zhang H, Hu B, Li Z, Du T, Shan J, Ye Z, et al. Pkcβii phosphorylates acsl4 to amplify lipid peroxidation to induce ferroptosis. Nat Cell Biol. 2022;24:88–98. 35027735 10.1038/s41556-021-00818-3

[44] Dai Y, Zhu C, Xiao W, Chen X, Cai Y. Mycobacterium tuberculosis induces host autophagic ferritin degradation for enhanced iron bioavailability and bacterial growth. Autophagy. 2024;20:943–5. 37198940 10.1080/15548627.2023.2213983 PMC11062391

[45] Muhoberac BB, Vidal R. Iron, ferritin, hereditary ferritinopathy, and neurodegeneration. Front Neurosci. 2019;13:1195. 31920471 10.3389/fnins.2019.01195 PMC6917665

[46] Gao M, Monian P, Quadri N, Ramasamy R, Jiang X. Glutaminolysis and transferrin regulate ferroptosis. Mol Cell. 2015;59:298–308. 26166707 10.1016/j.molcel.2015.06.011 PMC4506736

[47] Lin Y, Zhang Y, Huang T, Chen J, Li G, Zhang B, et al. Arginine deprivation induces quiescence and confers vulnerability to ferroptosis in colorectal cancer. Cancer Res. 2025;85:1663–79. 39992728 10.1158/0008-5472.CAN-24-1940 PMC12046318

[48] Werner A, Amann E, Schnitzius V, Habermeier A, Luckner-Minden C, Leuchtner N, et al. Induced arginine transport via cationic amino acid transporter-1 is necessary for human T-cell proliferation. Eur J Immunol. 2016;46:92–103. 26449889 10.1002/eji.201546047

[49] Zou Y, Henry WS, Ricq EL, Graham ET, Phadnis VV, Maretich P, et al. Plasticity of ether lipids promotes ferroptosis susceptibility and evasion. Nature. 2020;585:603–8. 32939090 10.1038/s41586-020-2732-8 PMC8051864

[50] Kang MJ, Fujino T, Sasano H, Minekura H, Yabuki N, Nagura H, et al. A novel arachidonate-preferring acyl-CoA synthetase is present in steroidogenic cells of the rat adrenal, ovary, and testis. Proc Natl Acad Sci USA. 1997;94:2880–4. 9096315 10.1073/pnas.94.7.2880 PMC20291

[51] Brown CW, Amante JJ, Goel HL, Mercurio AM. The α6β4 integrin promotes resistance to ferroptosis. J Cell Biol. 2017;216:4287–97. 28972104 10.1083/jcb.201701136 PMC5716272

[52] Kagan VE, Mao G, Qu F, Angeli JPF, Doll S, Croix CS, et al. Oxidized arachidonic and adrenic pes navigate cells to ferroptosis. Nat Chem Biol. 2017;13:81–90. 27842066 10.1038/nchembio.2238 PMC5506843

[53] Rivera A, Siracusa MC, Yap GS, Gause WC. Innate cell communication kick-starts pathogen-specific immunity. Nat Immunol. 2016;17:356–63. 27002843 10.1038/ni.3375 PMC4949486

[54] Mills CD, Kincaid K, Alt JM, Heilman MJ, Hill AM. M-1/m-2 macrophages and the Th1/Th2 paradigm. J Immunol. 2000;164:6166–73. 10843666 10.4049/jimmunol.164.12.6166

[55] Piccolo V, Curina A, Genua M, Ghisletti S, Simonatto M, Sabò A, et al. Opposing macrophage polarization programs show extensive epigenomic and transcriptional cross-talk. Nat Immunol. 2017;18:530–40. 28288101 10.1038/ni.3710 PMC5524187

[56] Gordon S, Martinez FO. Alternative activation of macrophages: mechanism and functions. Immunity. 2010;32:593–604. 20510870 10.1016/j.immuni.2010.05.007

[57] Gordon S. Alternative activation of macrophages. Nat Rev Immunol. 2003;3:23–35. 12511873 10.1038/nri978

[58] Sirén J, Pirhonen J, Julkunen I, Matikainen S. Ifn-alpha regulates tlr-dependent gene expression of ifn-alpha, ifn-beta, il-28, and il-29. J Immunol. 2005;174:1932–7. 15699120 10.4049/jimmunol.174.4.1932

[59] Ding L, Linsley PS, Huang LY, Germain RN, Shevach EM. IL-10 inhibits macrophage costimulatory activity by selectively inhibiting the up-regulation of B7 expression. J Immunol. 1993;151:1224–34. 7687627

[60] Yunna C, Mengru H, Lei W, Weidong C. Macrophage m1/m2 polarization. Eur J Pharmacol. 2020;877:173090. 32234529 10.1016/j.ejphar.2020.173090

[61] Gunassekaran GR, Poongkavithai Vadevoo SM, Baek M, Lee B. M1 macrophage exosomes engineered to foster M1 polarization and target the iL-4 receptor inhibit tumor growth by reprogramming tumor-associated macrophages into M1-like macrophages. Biomaterials. 2021;278:121137. 34560422 10.1016/j.biomaterials.2021.121137

[62] Sima C, Glogauer M. Macrophage subsets and osteoimmunology: tuning of the immunological recognition and effector systems that maintain alveolar bone. Periodontol 2000. 2013;63:80–101. 23931056 10.1111/prd.12032

[63] Fang J, Ou Q, Wu B, Li S, Wu M, Qiu J, et al. Tcpc inhibits M1 but promotes M2 macrophage polarization via regulation of the MAPK/nf-κb and Akt/STAT6 pathways in urinary tract infection. Cells 2022;11:2674. 10.3390/cells11172674 PMC9454685 36078080

[64] Rutschman R, Lang R, Hesse M, Ihle JN, Wynn TA, Murray PJ. Cutting edge: stat6-dependent substrate depletion regulates nitric oxide production. J Immunol. 2001;166:2173–7. 11160269 10.4049/jimmunol.166.4.2173

[65] Vodovotz Y, Bogdan C, Paik J, Xie QW, Nathan C. Mechanisms of suppression of macrophage nitric oxide release by transforming growth factor beta. J Exp Med. 1993;178:605–13. 7688028 10.1084/jem.178.2.605 PMC2191129

[66] Siebeler R, de Winther MPJ, Hoeksema MA. The regulatory landscape of macrophage interferon signaling in inflammation. J Allergy Clin Immunol. 2023;152:326–37. 37271317 10.1016/j.jaci.2023.04.022

[67] Wang S, Lu M, Wang W, Yu S, Yu R, Cai C, et al. Macrophage polarization modulated by nf-κb in polylactide membrane-treated peritendinous adhesion. Small. 2022;18:e2104112. 34816589 10.1002/smll.202104112

[68] Alexopoulou L, Holt AC, Medzhitov R, Flavell RA. Recognition of double-stranded RNA and activation of NF-kappaB by toll-like receptor 3. Nature. 2001;413:732–8. 11607032 10.1038/35099560

[69] Chao H, Zheng L, Hsu P, He J, Wu R, Xu S, et al. IL-13ra2 downregulation in fibroblasts promotes keloid fibrosis via Jak/Stat6 activation. JCI Insight 2023;8:e157091. 10.1172/jci.insight.157091 PMC10070111 36757802

[70] Zhang Z, Leng Z, Kang L, Yan X, Shi J, Ji Y, et al. Alcohol induces macrophage m2b polarization in colitis by modulating the TRPV1-MAPK/nf-κb pathways. Phytomedicine. 2024;130. 155580. 38810558 10.1016/j.phymed.2024.155580

[71] Lin Y, Wang Y, Peng Y, Liu F, Lin G, Huang S, et al. Interleukin 26 skews macrophage polarization towards M1 phenotype by activating cJun and the NF-κb pathway. Cells 2020;9:938. 10.3390/cells9040938 PMC7227026 32290250

[72] Liu Y, Wang L, Li S, Zhang T, Chen C, Hu J, et al. Mechanical stimulation improves rotator cuff tendon-bone healing via activating il-4/jak/Jak/Stat signaling pathway-mediated macrophage M2 polarization. J Orthop Transl. 2022;37:78–88. 10.1016/j.jot.2022.08.008 PMC9550856 36262964

[73] Cui Y, Chen C, Tang Z, Yuan W, Yue K, Cui P, et al. Trem2 deficiency aggravates renal injury by promoting macrophage apoptosis and polarization via the Jak-Stat pathway in mice. Cell Death Dis. 2024;15:401. 38849370 10.1038/s41419-024-06756-w PMC11161629

[74] Wang S, Liu R, Yu Q, Dong L, Bi Y, Liu G. Metabolic reprogramming of macrophages during infections and cancer. Cancer Lett. 2019;452:14–22. 30905817 10.1016/j.canlet.2019.03.015

[75] Ji L, Zhao X, Zhang B, Kang L, Song W, Zhao B, et al. Slc6a8-mediated creatine uptake and accumulation reprogram macrophage polarization via regulating cytokine responses. Immunity. 2019;51:272–84. 31399282 10.1016/j.immuni.2019.06.007

[76] Liu C, Hu F, Jiao G, Guo Y, Zhou P, Zhang Y, et al. Dental pulp stem cell-derived exosomes suppress M1 macrophage polarization through the ros-mapk-nfκb p65 signaling pathway after spinal cord injury. J Nanobiotechnol. 2022;20:65. 10.1186/s12951-022-01273-4 PMC8811988 35109874

[77] Bai X, Guo Y, Zhao Z, Li X, Dai D, Zhang J, et al. Macrophage polarization in cancer and beyond: from inflammatory signaling pathways to potential therapeutic strategies. Cancer Lett. 2025;625:217772. 40324582 10.1016/j.canlet.2025.217772

[78] Lawrence T, Natoli G. Transcriptional regulation of macrophage polarization: enabling diversity with identity. Nat Rev Immunol. 2011;11:750–61. 22025054 10.1038/nri3088

[79] Edsfeldt A, Swart M, Singh P, Dib L, Sun J, Cole JE, et al. Interferon regulatory factor-5-dependent CD11c+ macrophages contribute to the formation of rupture-prone atherosclerotic plaques. Eur Heart J. 2022;43:1864–77. 35567557 10.1093/eurheartj/ehab920 PMC9113304

[80] De Ioannes P, Escalante CR, Aggarwal AK. Structures of apo IRF-3 and IRF-7 DNA-binding domains: effect of loop L1 on DNA binding. Nucleic Acids Res. 2011;39:7300–7. 21596780 10.1093/nar/gkr325 PMC3167601

[81] Takaoka A, Yanai H, Kondo S, Duncan G, Negishi H, Mizutani T, et al. Integral role of IRF-5 in the gene induction programme activated by toll-like receptors. Nature. 2005;434:243–9. 15665823 10.1038/nature03308

[82] Lu H, Wu L, Liu L, Ruan Q, Zhang X, Hong W, et al. Quercetin ameliorates kidney injury and fibrosis by modulating m1/m2 macrophage polarization. Biochem Pharm. 2018;154:203–12. 29753749 10.1016/j.bcp.2018.05.007

[83] Yanai H, Chen H, Inuzuka T, Kondo S, Mak TW, Takaoka A, et al. Role of ifn regulatory factor 5 transcription factor in antiviral immunity and tumor suppression. Proc Natl Acad Sci USA. 2007;104:3402–7. 17360658 10.1073/pnas.0611559104 PMC1805533

[84] Heinz LX, Lee J, Kapoor U, Kartnig F, Sedlyarov V, Papakostas K, et al. Tasl is the slc15a4-associated adaptor for Irf5 activation by TLR7-9. Nature. 2020;581:316–22. 32433612 10.1038/s41586-020-2282-0 PMC7610944

[85] Huang C, Wang J, Liu H, Huang R, Yan X, Song M, et al. Ketone body β-hydroxybutyrate ameliorates colitis by promoting m2 macrophage polarization through the Stat6-dependent signaling pathway. BMC Med. 2022;20:148. 35422042 10.1186/s12916-022-02352-x PMC9011974

[86] Abdelaziz MH, Abdelwahab SF, Wan J, Cai W, Huixuan W, Jianjun C, et al. Alternatively activated macrophages: a double-edged sword in allergic asthma. J Transl Med. 2020;18:58. 32024540 10.1186/s12967-020-02251-w PMC7003359

[87] Hsu C, Pan Y, Zheng Y, Lo RY, Yang F. Ultrasound reduces inflammation by modulating m1/m2 polarization of microglia through stat1/stat6/pparγ signaling pathways. CNS Neurosci Ther. 2023;29:4113–23. 37401041 10.1111/cns.14333 PMC10651950

[88] Tu Y, Liu J, Kong D, Guo X, Li J, Long Z, et al. Irisin drives macrophage anti-inflammatory differentiation via Jak2-Stat6-dependent activation of pparγ and Nrf2 signaling. Free Radic Biol Med. 2023;201:98–110. 36940733 10.1016/j.freeradbiomed.2023.03.014

[89] Ding Y, Sun Y, Wang H, Zhao H, Yin R, Zhang M, et al. Atherosis-associated lnc_000048 activates pkr to enhance stat1-mediated polarization of THP-1 macrophages to M1 phenotype. Neural Regen Res. 2024;19:2488–98. 38526285 10.4103/NRR.NRR-D-23-01355 PMC11090429

[90] Somensi N, Rabelo TK, Guimarães AG, Quintans-Junior LJ, de Souza Araújo AA, Moreira JCF, et al. Carvacrol suppresses LPS-induced pro-inflammatory activation in RAW 264.7 macrophages through erk1/2 and nf-kb pathway. Int Immunopharmacol. 2019;75:105743. 31357087 10.1016/j.intimp.2019.105743

[91] de Carvalho TG, Lara P, Jorquera-Cordero C, Aragão CFS, de Santana Oliveira A, Garcia VB, et al. Inhibition of murine colorectal cancer metastasis by targeting m2-tam through Stat3/NF-kb/Akt signaling using macrophage 1-derived extracellular vesicles loaded with oxaliplatin, retinoic acid, and Libidibia ferrea. Biomed Pharmacother. 2023;168. 115663. 37832408 10.1016/j.biopha.2023.115663

[92] Wu J, Feng Z, Chen L, Li Y, Bian H, Geng J, et al. Tnf antagonist sensitizes synovial fibroblasts to ferroptotic cell death in collagen-induced arthritis mouse models. Nat Commun. 2022;13:676. 35115492 10.1038/s41467-021-27948-4 PMC8813949

[93] Udalova IA, Mantovani A, Feldmann M. Macrophage heterogeneity in the context of rheumatoid arthritis. Nat Rev Rheumatol. 2016;12:472–85. 27383913 10.1038/nrrheum.2016.91

[94] Luo L, Huang F, Zhong S, Ding R, Su J, Li X. Astaxanthin attenuates ferroptosis via Keap1-Nrf2/ho-1 signaling pathways in LPS-induced acute lung injury. Life Sci. 2022;311:121091. 36252699 10.1016/j.lfs.2022.121091

[95] Dai E, Han L, Liu J, Xie Y, Zeh HJ, Kang R, et al. Ferroptotic damage promotes pancreatic tumorigenesis through a tmem173/sting-dependent DNA sensor pathway. Nat Commun. 2020;11:6339. 33311482 10.1038/s41467-020-20154-8 PMC7732843

[96] Ling X, Wei S, Ling D, Cao S, Chang R, Wang Q, et al. Irf7 regulates the expression of srg3 and ferroptosis axis aggravated sepsis-induced acute lung injury. Cell Mol Biol Lett. 2023;28:91. 37946128 10.1186/s11658-023-00495-0 PMC10634032

[97] Ling Y, Yang Y, Ren N, Xu H, Cheng C, Lu D, et al. Jinwu jiangu capsule attenuates rheumatoid arthritis via the slc7a11/gsh/gpx4 pathway in M1 macrophages. Phytomedicine. 2024;135:156232. 39547097 10.1016/j.phymed.2024.156232

[98] Xiong H, Zhai Y, Meng Y, Wu Z, Qiu A, Cai Y, et al. Acidosis activates breast cancer ferroptosis through zfand5/slc3a2 signaling axis and elicits m1 macrophage polarization. Cancer Lett. 2024;587:216732. 38360142 10.1016/j.canlet.2024.216732

[99] Jiang Q, Qiu Y, Kurland IJ, Drlica K, Subbian S, Tyagi S, et al. Glutamine is required for M1-like polarization of macrophages in response to Mycobacterium tuberculosis infection. mBio. 2022;13:e127422. 10.1128/mbio.01274-22 PMC9426538 35762591

[100] Xiao Q, Hou R, Xie L, Niu M, Pan X, Zhu X. Macrophage metabolic reprogramming and atherosclerotic plaque microenvironment: fostering each other? Clin Transl Med. 2023;13:e1257. 37151159 10.1002/ctm2.1257 PMC10165242

[101] Vats D, Mukundan L, Odegaard JI, Zhang L, Smith KL, Morel CR, et al. Oxidative metabolism and PGC-1β attenuate macrophage-mediated inflammation. Cell Metab. 2006;4:13–24. 16814729 10.1016/j.cmet.2006.05.011 PMC1904486

[102] Murray PJ. Macrophage polarization. Annu Rev Physiol. 2017;79:541–66. 27813830 10.1146/annurev-physiol-022516-034339

[103] Kapralov AA, Yang Q, Dar HH, Tyurina YY, Anthonymuthu TS, Kim R, et al. Redox lipid reprogramming commands susceptibility of macrophages and microglia to ferroptotic death. Nat Chem Biol. 2020;16:278–90. 32080625 10.1038/s41589-019-0462-8 PMC7233108

[104] Hu X, Ma Z, Xu B, Li S, Yao Z, Liang B, et al. Glutamine metabolic microenvironment drives m2 macrophage polarization to mediate trastuzumab resistance in HER2-positive gastric cancer. Cancer Commun. 2023;43:909–37. 10.1002/cac2.12459 PMC10397568 37434399

[105] Zhu Y, Zhang S, Sun J, Wang T, Liu Q, Wu G, et al. Cigarette smoke promotes oral leukoplakia via regulating glutamine metabolism and m2 polarization of macrophages. Int J Oral Sci. 2021;13:25. 34373444 10.1038/s41368-021-00128-2 PMC8352977

[106] Zhu Y, Chen X, Lu Y, Xia L, Fan S, Huang Q, et al. Glutamine mitigates murine burn sepsis by supporting macrophage m2 polarization through repressing the sirt5-mediated desuccinylation of pyruvate dehydrogenase. Burns Trauma. 2022;10:tkac41. 10.1093/burnst/tkac041 PMC9801296 36601059

[107] Yang M, Shen Z, Zhang X, Song Z, Zhang Y, Lin Z, et al. Ferroptosis of macrophages facilitates bone loss in apical periodontitis via Nrf2/fsp1/ROS pathway. Free Radic Biol Med. 2023;208:334–47. 37619958 10.1016/j.freeradbiomed.2023.08.020

[108] Luo X, Wang Y, Zhu X, Chen Y, Xu B, Bai X, et al. Mcl attenuates atherosclerosis by suppressing macrophage ferroptosis via targeting Keap1/Nrf2 interaction. Redox Biol. 2024;69:102987. 38100883 10.1016/j.redox.2023.102987 PMC10761782

[109] Bao X, Luo X, Bai X, Lv Y, Weng X, Zhang S, et al. Cigarette tar mediates macrophage ferroptosis in atherosclerosis through the hepcidin/fpn/slc7a11 signaling pathway. Free Radic Biol Med. 2023;201:76–88. 36933812 10.1016/j.freeradbiomed.2023.03.006

[110] Feng Z, Meng F, Huo F, Zhu Y, Qin Y, Gui Y, et al. Inhibition of ferroptosis rescues m2 macrophages and alleviates arthritis by suppressing the HMGB1/TLR4/Stat3 axis in m1 macrophages. Redox Biol. 2024;75:103255. 39029270 10.1016/j.redox.2024.103255 PMC11304870

[111] Newton K. Ripk1 and Ripk3: critical regulators of inflammation and cell death. Trends Cell Biol. 2015;25:347–53. 25662614 10.1016/j.tcb.2015.01.001

[112] Bian W, Wei L, Wang K. Carthamin yellow alleviates dextran sodium sulfate-induced ulcerative colitis by repairing the intestinal barrier and activating the Nrf2/GPX4 axis. Int Immunopharmacol. 2024;141:113020. 39208524 10.1016/j.intimp.2024.113020

[113] Kenneth NS, Mudie S, Naron S, Rocha S. Tfr1 interacts with the IKK complex and is involved in ikk-nf-κb signalling. Biochem J. 2013;449:275–84. 23016877 10.1042/BJ20120625 PMC3537175

[114] He Y, Hara H, Núñez G. Mechanism and regulation of NLRP3 inflammasome activation. Trends Biochem Sci. 2016;41:1012–21. 27669650 10.1016/j.tibs.2016.09.002 PMC5123939

[115] Zhang J, Liu X, Wan C, Liu Y, Wang Y, Meng C, et al. Nlrp3 inflammasome mediates M1 macrophage polarization and il-1β production in inflammatory root resorption. J Clin Periodontol. 2020;47:451–60. 31976565 10.1111/jcpe.13258

[116] Franke M, Bieber M, Kraft P, Weber ANR, Stoll G, Schuhmann MK. The NLRP3 inflammasome drives inflammation in ischemia/reperfusion injury after transient middle cerebral artery occlusion in mice. Brain Behav Immun. 2021;92:223–33. 33307174 10.1016/j.bbi.2020.12.009

[117] Gao Y, Mi N, Wu W, Zhao Y, Fan F, Liao W, et al. Transfer of inflammatory mitochondria via extracellular vesicles from m1 macrophages induces ferroptosis of pancreatic beta cells in acute pancreatitis. J Extracell Vesicles. 2024;13:e12410. 38320981 10.1002/jev2.12410 PMC10847061

[118] Park E, Chung SW. ROS-mediated autophagy increases intracellular iron levels and ferroptosis by ferritin and transferrin receptor regulation. Cell Death Dis. 2019;10:822. 31659150 10.1038/s41419-019-2064-5 PMC6817894

[119] Dewan A, Jain C, Das M, Tripathi A, Sharma AK, Singh H, et al. Intracellular peroxynitrite perturbs redox balance, bioenergetics, and Fe-S cluster homeostasis in Mycobacterium tuberculosis. Redox Biol. 2024;75:103285. 39128229 10.1016/j.redox.2024.103285 PMC11369450

[120] Riobó NA, Clementi E, Melani M, Boveris A, Cadenas E, Moncada S, et al. Nitric oxide inhibits mitochondrial NADH: ubiquinone reductase activity through peroxynitrite formation. Biochem J. 2001;359:139–45. 11563977 10.1042/0264-6021:3590139 PMC1222129

[121] Yao T, Dong X, Lv J, Fu L, Li L. Propionate alleviated colitis by modulating iron homeostasis to inhibit ferroptosis and macrophage polarization. Int Immunopharmacol. 2025;162:115151. 40609203 10.1016/j.intimp.2025.115151

[122] Liao J, Wei M, Wang J, Zeng J, Liu D, Du Q, et al. Naotaifang formula attenuates ogd/r-induced inflammation and ferroptosis by regulating microglial M1/M2 polarization through BMP6/Smad signaling pathway. Biomed Pharmacother. 2023;167. 115465. 37713988 10.1016/j.biopha.2023.115465

[123] Thompson K, Menzies S, Muckenthaler M, Torti FM, Wood T, Torti SV, et al. Mouse brains deficient in h-ferritin have normal iron concentration but a protein profile of iron deficiency and increased evidence of oxidative stress. J Neurosci Res. 2003;71:46–63. 12478613 10.1002/jnr.10463

[124] Chen X, Wan Z, Yang L, Song S, Fu Z, Tang K, et al. Exosomes derived from reparative m2-like macrophages prevent bone loss in murine periodontitis models via IL-10 mRNA. J Nanobiotechnol. 2022;20:110. 10.1186/s12951-022-01314-y PMC8898524 35248085

[125] Jung M, Ma Y, Iyer RP, Deleon-Pennell KY, Yabluchanskiy A, Garrett MR, et al. IL-10 improves cardiac remodeling after myocardial infarction by stimulating M2 macrophage polarization and fibroblast activation. Basic Res Cardiol. 2017;112:33. 28439731 10.1007/s00395-017-0622-5 PMC5575998

[126] Wu W, Luo Z, Shen D, Lan T, Xiao Z, Liu M, et al. IL-10 protects against OPC ferroptosis by regulating lipid reactive oxygen species levels post stroke. Redox Biol. 2024;69:102982. 38070317 10.1016/j.redox.2023.102982 PMC10755589

[127] Xu S, Chen Y, Miao J, Li Y, Liu J, Zhang J, et al. Esculin inhibits hepatic stellate cell activation and CCL(4)-induced liver fibrosis by activating the Nrf2/GPX4 signaling pathway. Phytomedicine. 2024;128:155465. 38471319 10.1016/j.phymed.2024.155465

[128] Chen Y, Dai Y, Huang Y, Zhang L, Zhang C, Gao H, et al. Inhibition of tubular epithelial cells ferroptosis alleviates renal interstitial fibrosis by reducing lipid hydroperoxides and tgf-β/smad signaling. Cell Commun Signal. 2025;23:81. 39934851 10.1186/s12964-025-02068-4 PMC11817721

[129] Man S, Ma W, Jiang H, Haider A, Shi S, Li X, et al. Evaluating the efficacy and mechanisms of hua-zhuo-ning-fu-decoction on psoriasis using integrated bioinformatics analysis and metabolomics. J Ethnopharmacol. 2024;325:117856. 38316220 10.1016/j.jep.2024.117856

[130] Yang L, Ye F, Liu J, Klionsky DJ, Tang D, Kang R. Extracellular sqstm1 exacerbates acute pancreatitis by activating autophagy-dependent ferroptosis. Autophagy. 2023;19:1733–44. 36426912 10.1080/15548627.2022.2152209 PMC10262765

[131] Yang T, Wu P, Jiang L, Chen R, Jin Q, Ye G. Cardamonin attenuates myocardial ischemia/reperfusion-induced ferroptosis through promoting Stat3 signaling. J Inflamm Res. 2024;17:8861–79. 39569022 10.2147/JIR.S486412 PMC11577436

[132] Dai E, Han L, Liu J, Xie Y, Kroemer G, Klionsky DJ, et al. Autophagy-dependent ferroptosis drives tumor-associated macrophage polarization via release and uptake of oncogenic Kras protein. Autophagy. 2020;16:2069–83. 31920150 10.1080/15548627.2020.1714209 PMC7595620

[133] Yang M, Chen X, Hu X, Li H, Huang H, Fang Y, et al. The nf-κb-slc7a11 axis regulates ferroptosis sensitivity in inflammatory macrophages. Cell Insight. 2025;4:100257. 40677785 10.1016/j.cellin.2025.100257 PMC12268560

[134] Xiang X, Wang J, Lu D, Xu X. Targeting tumor-associated macrophages to synergize tumor immunotherapy. Signal Transduct Target Ther. 2021;6:75. 33619259 10.1038/s41392-021-00484-9 PMC7900181

[135] Cui K, Wang K, Huang Z. Ferroptosis and the tumor microenvironment. J Exp Clin Cancer Res. 2024;43:315. 39614322 10.1186/s13046-024-03235-0 PMC11607824

[136] Vitale I, Manic G, Coussens LM, Kroemer G, Galluzzi L. Macrophages and metabolism in the tumor microenvironment. Cell Metab. 2019;30:36–50. 31269428 10.1016/j.cmet.2019.06.001

[137] Rojas A, Delgado-López F, Pérez-Castro R, González I, Romero J, Rojas I, et al. Hmgb1 enhances the protumoral activities of m2 macrophages by a rage-dependent mechanism. Tumour Biol. 2016;37:3321–9. 26440051 10.1007/s13277-015-3940-y

[138] Ma J, Zhang H, Chen Y, Liu X, Tian J, Shen W. The role of macrophage iron overload and ferroptosis in atherosclerosis. Biomolecules 2022;12:1702. 10.3390/biom12111702 PMC9688033 36421722

[139] House IG, Savas P, Lai J, Chen AXY, Oliver AJ, Teo ZL, et al. Macrophage-derived CXCL9 and CXCL10 are required for antitumor immune responses following immune checkpoint blockade. Clin Cancer Res. 2020;26:487–504. 31636098 10.1158/1078-0432.CCR-19-1868

[140] Luo X, Gong H, Li Z, Li D, Li Z, Sun J, et al. Phospholipid peroxidation in macrophages confers tumor resistance by suppressing phagocytic capability towards ferroptotic cells. Cell Death Differ. 2024;31:1184–201. 39103535 10.1038/s41418-024-01351-0 PMC11369141

[141] Do MH, Shi W, Ji L, Ladewig E, Zhang X, Srivastava RM, et al. Reprogramming tumor-associated macrophages to outcompete endovascular endothelial progenitor cells and suppress tumor neoangiogenesis. Immunity. 2023;56:2555–69. 37967531 10.1016/j.immuni.2023.10.010 PMC11284818

[142] Sun X, Gao D, Gao L, Zhang C, Yu X, Jia B, et al. Molecular imaging of tumor-infiltrating macrophages in a preclinical mouse model of breast cancer. Theranostics. 2015;5:597–608. 25825599 10.7150/thno.11546 PMC4377728

[143] Li H, Yang P, Wang J, Zhang J, Ma Q, Jiang Y, et al. Hlf regulates ferroptosis, development and chemoresistance of triple-negative breast cancer by activating tumor cell-macrophage crosstalk. J Hematol Oncol. 2022;15:2. 34991659 10.1186/s13045-021-01223-x PMC8740349

[144] Arwert EN, Harney AS, Entenberg D, Wang Y, Sahai E, Pollard JW, et al. A unidirectional transition from migratory to perivascular macrophage is required for tumor cell intravasation. Cell Rep. 2018;23:1239–48. 29719241 10.1016/j.celrep.2018.04.007 PMC5946803

[145] Mantovani A, Sozzani S, Locati M, Allavena P, Sica A. Macrophage polarization: tumor-associated macrophages as a paradigm for polarized M2 mononuclear phagocytes. Trends Immunol. 2002;23:549–55. 12401408 10.1016/s1471-4906(02)02302-5

[146] Huang J, Pan H, Sun J, Wu J, Xuan Q, Wang J, et al. Tmem147 aggravates the progression of HCC by modulating cholesterol homeostasis, suppressing ferroptosis, and promoting the m2 polarization of tumor-associated macrophages. J Exp Clin Cancer Res. 2023;42:286. 37891677 10.1186/s13046-023-02865-0 PMC10612308

[147] Fan C, Chen C, Chen L, Chua KV, Hung H, Hsu JT, et al. Extracellular hsp90α induces myd88-irak complex-associated ikkα/β-nf-κb/irf3 and jak2/tyk2-stat-3 signaling in macrophages for tumor-promoting m2-polarization. Cells 2022;11:229. 10.3390/cells11020229 PMC8774043 35053345

[148] Tang B, Zhu J, Wang Y, Chen W, Fang S, Mao W, et al. Targeted xct-mediated ferroptosis and protumoral polarization of macrophages is effective against HCC and enhances the efficacy of the anti-pd-1/l1 response. Adv Sci. 2023;10:e2203973. 10.1002/advs.202203973 PMC9839855 36442849

[149] Zheng N, Li F, Huang Q, Huang X, Maj T. Macrophages and macrophage extracellular vesicles confer cancer ferroptosis resistance via prdx6-mediated mitophagy inhibition. Redox Biol. 2025;86:103826. 40825268 10.1016/j.redox.2025.103826 PMC12391288

[150] Kainulainen K, Takabe P, Heikkinen S, Aaltonen N, de la Motte C, Rauhala L, et al. M1 macrophages induce protumor inflammation in melanoma cells through tnfr-nf-κb signaling. J Investig Dermatol. 2022;142:3041–51. 35580697 10.1016/j.jid.2022.04.024

[151] Luo Y, Tian G, Fang X, Bai S, Yuan G, Pan Y. Ferroptosis and its potential role in glioma: from molecular mechanisms to therapeutic opportunities. Antioxidants. 2022;11:2123. 10.3390/antiox11112123 PMC9686959 36358495

[152] Cutolo M, Campitiello R, Gotelli E, Soldano S. The role of M1/M2 macrophage polarization in rheumatoid arthritis synovitis. Front Immunol. 2022;13:867260. 35663975 10.3389/fimmu.2022.867260 PMC9161083

[153] Lu X, Li L, Lin J, Wu X, Li W, Tan C, et al. Paarh promotes M2 macrophage polarization and immune evasion of liver cancer cells through Vegf protein. Int J Biol Macromol. 2024;281:136580. 39406326 10.1016/j.ijbiomac.2024.136580

[154] Wang Y, Liu Y, Long M, Dong Y, Li L, Zhou X. Nanoparticles target m2 macrophages to silence kallikrein-related peptidase 12 for the treatment of tuberculosis and drug-resistant tuberculosis. Acta Biomater. 2024;188:358–73. 39305944 10.1016/j.actbio.2024.09.026

[155] Zhang D, Man D, Lu J, Jiang Y, Ding B, Su R, et al. Mitochondrial tspo promotes hepatocellular carcinoma progression through ferroptosis inhibition and immune evasion. Adv Sci. 2023;10:e2206669. 10.1002/advs.202206669 PMC10214260 36994647

[156] Zhou Q, Yu H, Chen Y, Ren J, Lu Y, Sun Y. The crl3(kctd10) ubiquitin ligase-usp18 axis coordinately regulates cystine uptake and ferroptosis by modulating slc7a11. Proc Natl Acad Sci USA. 2024;121:e1974312175. 10.1073/pnas.2320655121 PMC11252818 38959043

[157] Shi P, Hu B, Liu Y, Li X, Li W, Huang C, et al. Long non-coding RNA norad suppresses erastin-induced ferroptosis in breast cancer by upregulating slc7a11 via targeting the fus/nr3c1 axis: experimental studies. Int J Surg. 2025;111:4295–313. 40405780 10.1097/JS9.0000000000002531

[158] Arora S, Singh P, Ahmad S, Ahmad T, Dohare R, Almatroodi SA, et al. Comprehensive integrative analysis reveals the association of KLF4 with macrophage infiltration and polarization in the lung cancer microenvironment. Cells 2021;10:2091. 10.3390/cells10082091 PMC8392240 34440860

[159] Gu Z, Liu T, Liu C, Yang Y, Tang J, Song H, et al. Ferroptosis-strengthened metabolic and inflammatory regulation of tumor-associated macrophages provokes potent tumoricidal activities. Nano Lett. 2021;21:6471–9. 34292757 10.1021/acs.nanolett.1c01401

[160] Gao S, Zhou J, Liu N, Wang L, Gao Q, Wu Y, et al. Curcumin induces m2 macrophage polarization by secretion iL-4 and/or iL-13. J Mol Cell Cardiol. 2015;85:131–9. 25944087 10.1016/j.yjmcc.2015.04.025

[161] Wang M, Jin L, Zhang Q, Zhu W, He H, Lou S, et al. Curcumin analog JM-2 alleviates diabetic cardiomyopathy inflammation and remodeling by inhibiting the nf-κb pathway. Biomed Pharmacother. 2022;154:113590. 36057221 10.1016/j.biopha.2022.113590

[162] Kioi M, Vogel H, Schultz G, Hoffman RM, Harsh GR, Brown JM. Inhibition of vasculogenesis, but not angiogenesis, prevents the recurrence of glioblastoma after irradiation in mice. J Clin Investig. 2010;120:694–705. 20179352 10.1172/JCI40283 PMC2827954

[163] Kinaret PAS, Scala G, Federico A, Sund J, Greco D. Carbon nanomaterials promote m1/m2 macrophage activation. Small. 2020;16:e1907609. 32250056 10.1002/smll.201907609

[164] Lu Q, Kou D, Lou S, Ashrafizadeh M, Aref AR, Canadas I, et al. Nanoparticles in tumor microenvironment remodeling and cancer immunotherapy. J Hematol Oncol. 2024;17:16. 38566199 10.1186/s13045-024-01535-8 PMC10986145

[165] Liao P, Wang W, Wang W, Kryczek I, Li X, Bian Y, et al. Cd8(+) t cells and fatty acids orchestrate tumor ferroptosis and immunity via ACSL4. Cancer Cell. 2022;40:365–78. 35216678 10.1016/j.ccell.2022.02.003 PMC9007863

[166] Zeki J, Yavuz B, Wood L, Shimada H, Kaplan DL, Chiu B. Concurrent application of interferon-gamma and vincristine inhibits tumor growth in an orthotopic neuroblastoma mouse model. Pediatr Surg Int. 2023;39:241. 37500800 10.1007/s00383-023-05523-w

[167] Wang H, Zhang H, Chen Y, Wang H, Tian Y, Yi X, et al. Targeting wnt/β-catenin signaling exacerbates ferroptosis and increases the efficacy of melanoma immunotherapy via the regulation of mitf. Cells 2022;11:3580. 10.3390/cells11223580 PMC9688625 36429010

[168] Xiang J, Suo M, Lan J, Ning S, Liu H, Hong W, et al. Novel carrier-free nanomedicine for regulating macrophage phenotype to amplify anti-tumor photoimmunotherapy. Small. 2025;21:e5304. 10.1002/smll.202505304 40746212

[169] Zhang C, Weng Y, Wang H, Zhan S, Li C, Zheng D, et al. A synergistic effect of triptolide and curcumin on rheumatoid arthritis by improving cell proliferation and inducing cell apoptosis via inhibition of the IL-17/nf-κb signaling pathway. Int Immunopharmacol. 2024;142:112953. 39226828 10.1016/j.intimp.2024.112953

[170] Xu Q, Zhang M, Huang Q, Gao S, Chu S, Li Q, et al. Organic aie nanoradiosensitizer potentiates x-ray-triggered continuous reactive oxygen species generation for potent cancer radioimmunotherapy. Adv Mater. 2025;37:e2502898. 40534404 10.1002/adma.202502898 PMC12411993

[171] Zhao Y, Wang Q, Zhu J, Cai J, Feng X, Song Q, et al. Identification of kw-2449 as a dual inhibitor of ferroptosis and necroptosis reveals that autophagy is a targetable pathway for necroptosis inhibitors to prevent ferroptosis. Cell Death Dis. 2024;15:764. 39433736 10.1038/s41419-024-07157-9 PMC11493980

[172] Ma Y, Zhang Q, Li D, Zhao K, Li Z, Liu Y, et al. Intercellular propagation of Ripk1/Ripk3 amyloid fibrils. Proc Natl Acad Sci USA. 2025;122. e1787939174. 10.1073/pnas.2507028122 PMC12478036 40956882

[173] Ridker PM, Rane M. Interleukin-6 signaling and anti-interleukin-6 therapeutics in cardiovascular disease. Circ Res. 2021;128:1728–46. 33998272 10.1161/CIRCRESAHA.121.319077

[174] Hsu S, Li C, Lin I, Syue W, Chen Y, Cheng K, et al. Inflammation-related pyroptosis, a novel programmed cell death pathway, and its crosstalk with immune therapy in cancer treatment. Theranostics. 2021;11:8813–35. 34522213 10.7150/thno.62521 PMC8419056

[175] Li N, Wang W, Zhou H, Wu Q, Duan M, Liu C, et al. Ferritinophagy-mediated ferroptosis is involved in sepsis-induced cardiac injury. Free Radic Biol Med. 2020;160:303–18. 32846217 10.1016/j.freeradbiomed.2020.08.009

[176] Wang S, Liu C, Pan S, Miao Q, Xue J, Xun J, et al. Deferoxamine attenuates lipopolysaccharide-induced inflammatory responses and protects against endotoxic shock in mice. Biochem Biophys Res Commun. 2015;465:305–11. 26277391 10.1016/j.bbrc.2015.08.032

[177] Friedmann Angeli JP, Schneider M, Proneth B, Tyurina YY, Tyurin VA, Hammond VJ, et al. Inactivation of the ferroptosis regulator gpx4 triggers acute renal failure in mice. Nat Cell Biol. 2014;16:1180–91. 25402683 10.1038/ncb3064 PMC4894846

[178] Viswanathan VS, Ryan MJ, Dhruv HD, Gill S, Eichhoff OM, Seashore-Ludlow B, et al. Dependency of a therapy-resistant state of cancer cells on a lipid peroxidase pathway. Nature. 2017;547:453–7. 28678785 10.1038/nature23007 PMC5667900

[179] Li Q, Sun J, Mohammadtursun N, Wu J, Dong J, Li L. Curcumin inhibits cigarette smoke-induced inflammation via modulating the pparγ-nf-κb signaling pathway. Food Funct. 2019;10:7983–94. 31773117 10.1039/c9fo02159k

[180] Li L, Jiang W, Yu B, Liang H, Mao S, Hu X, et al. Quercetin improves cerebral ischemia/reperfusion injury by promoting microglia/macrophages m2 polarization via regulating PI3K/AKT/nf-κb signaling pathway. Biomed Pharmacother. 2023;168. 115653. 37812891 10.1016/j.biopha.2023.115653

[181] Deng C, Huo M, Chu H, Zhuang X, Deng G, Li W, et al. Exosome circatp8a1 induces macrophage M2 polarization by regulating the miR-1-3p/STAT6 axis to promote gastric cancer progression. Mol Cancer. 2024;23. 49. 38459596 10.1186/s12943-024-01966-4 PMC10921793

[182] Puylaert P, Roth L, Van Praet M, Pintelon I, Dumitrascu C, van Nuijs A, et al. Effect of erythrophagocytosis-induced ferroptosis during angiogenesis in atherosclerotic plaques. Angiogenesis. 2023;26:505–22. 37120604 10.1007/s10456-023-09877-6 PMC10542744

[183] Bai T, Li M, Liu Y, Qiao Z, Wang Z. Inhibition of ferroptosis alleviates atherosclerosis through attenuating lipid peroxidation and endothelial dysfunction in mouse aortic endothelial cells. Free Radic Biol Med. 2020;160:92–102. 32768568 10.1016/j.freeradbiomed.2020.07.026

[184] Ma Y, Mouton AJ, Lindsey ML. Cardiac macrophage biology in the steady-state heart, the aging heart, and following myocardial infarction. Transl Res. 2018;191:15–28. 29106912 10.1016/j.trsl.2017.10.001 PMC5846093

[185] Ding T, Song M, Wu Y, Li Z, Zhang S, Fan X. Schisandrin b ameliorates alzheimer’s disease by suppressing neuronal ferroptosis and ensuing microglia M1 polarization. Phytomedicine. 2025;142:156780. 40382817 10.1016/j.phymed.2025.156780

[186] Park MW, Cha HW, Kim J, Kim JH, Yang H, Yoon S, et al. Nox4 promotes ferroptosis of astrocytes by oxidative stress-induced lipid peroxidation via the impairment of mitochondrial metabolism in alzheimer’s disease. Redox Biol. 2021;41:101947. 33774476 10.1016/j.redox.2021.101947 PMC8027773

[187] Yu H, Chang Q, Sun T, He X, Wen L, An J, et al. Metabolic reprogramming and polarization of microglia in parkinson’s disease: role of inflammasome and iron. Ageing Res Rev. 2023;90:102032. 37572760 10.1016/j.arr.2023.102032

[188] Zhu L, Ma L, Du X, Jiang Y, Gao J, Fan Z, et al. M2 microglia-derived exosomes protect against glutamate-induced HT22 cell injury via exosomal mir-124-3p. Mol Neurobiol. 2024;61:7845–61. 38433165 10.1007/s12035-024-04075-x PMC11415474

[189] Long Q, Li T, Zhu Q, He L, Zhao B. Suanzaoren decoction alleviates neuronal loss, synaptic damage and ferroptosis via activating dj-1/nrf2 signaling pathway. J Ethnopharmacol. 2024;323:117679. 38160863 10.1016/j.jep.2023.117679

[190] Zhong X, Gong S, Meng L, Yao W, Du K, Jiao L, et al. Cordycepin modulates microglial m2 polarization coupled with mitochondrial metabolic reprogramming by targeting HKII and PDK2. Adv Sci. 2024;11. e2304687. 10.1002/advs.202304687 PMC11336950 38889331

[191] Zhang Y, Chen J, Zheng J, Cheng Y, Weng W, Zhong R, et al. Pterosin b improves cognitive dysfunction by promoting microglia M1/M2 polarization through inhibiting the KLF5/PARP14 pathway. Phytomedicine. 2024;135. 156152. 39413455 10.1016/j.phymed.2024.156152

[192] Tao W, Hu Y, Chen Z, Dai Y, Hu Y, Qi M. Magnolol attenuates depressive-like behaviors by polarizing microglia towards the m2 phenotype through the regulation of Nrf2/ho-1/nlrp3 signaling pathway. Phytomedicine. 2021;91:153692. 34411834 10.1016/j.phymed.2021.153692

[193] Li H, Cao Z, Liu C, Wang Y, Wang L, Tang Y, et al. Quercetin inhibits neuronal pyroptosis and ferroptosis by modulating microglial m1/m2 polarization in atherosclerosis. J Agric Food Chem. 2024;72:12156–70. 38755521 10.1021/acs.jafc.4c01134

